# Two-step ultrasonic cavitation controlled delivery of brain exogenous nucleic acids for ischemic stroke using acoustic-cationic-polymeric-nanodroplets

**DOI:** 10.1007/s13346-025-01828-6

**Published:** 2025-03-06

**Authors:** Wei Dong, Guihu Wang, Yichao Chai, Wenjuan Li, Shichang Liu, Huasheng Liu, Wenlei Guo, Senyang Li, Xinrui He, Mingxi Wan, Zongfang Li, Yujin Zong

**Affiliations:** 1 National-Local Joint Engineering Research Center of Biodiagnosis & Biotherapy, The Second Affiliated Hospital of Xi’an Jiaotong University, Xi’an, China; 2 Key Laboratory of Biomedical Information Engineering of Ministry of Education, Department of Biomedical Engineering, School of Life Science and Technology, Xi’an Jiaotong University, Xi’an, China; 3 Department of Spine Surgery, Honghui Hospital, Xi’an Jiaotong University, Xi’an, China; 4 Department of Hematology, The First Affiliated Hospital, Xi’an Jiaotong University, Xi’an, China

**Keywords:** Two-step ultrasonic cavitation, Acoustic cationic polymeric nanodroplets, Controlled delivery of exogenous nucleic acids for ischemic stroke, Sonoporation, BBB opening

## Abstract

Inefficient and low-precision delivery of exogenous nucleic acids (ENA) severely limits gene therapy on ischemic stroke (IS). Two problems need to be urgently addressed to improve the efficacy of gene therapy; first, the blood brain barrier (BBB) should be open to promote the accumulation of ENA or genetic material carriers in the ischemic brain parenchyma, and second, the efficient delivery of ENA into the ischemic cells. Previous studies applied ultrasonic cavitation either for opening BBB or for inducing sonoporation to deliver genetic materials into cells. However, the effectiveness of the two-step ultrasonic cavitation to deliver ENA in the brain remains unclear, let alone the genetic materials to be controllably delivered into the ischemic brain parenchyma of the IS. This study systematically explored the BBB opening and ENA delivery by the two-step ultrasonic cavitation using artificial acoustic-cationic-polymeric-nanodroplets (ACPNs). The results demonstrated that the first focused ultrasound (FUS), set at parameters of 3.3 MPa, 20 Hz, 200 cycles and 5 s, stimulating intravascular ACPNs cavitation effectively opened BBB to allow nonactivated ACPN extravasation and accumulation into the ischemic brain parenchyma. Then, the extravascular ACPNs enhanced the second ultrasonic cavitation that noninvasively and efficiently controlled ENA delivery to the ischemic cells through sonoporation, particularly applying 3.3 MPa, 60 Hz, 200 cycles and 9 s to control FAM-eNA delivery, and 3.6 MPa, 20 Hz, 200 cycles and 7 s for pEGFP-C1 controlled delivery. Overall, the two-step ultrasonic cavitation represented a potential strategy for IS-targeted ENA controlled delivery.

**Graphical abstract:**

**Figure:**
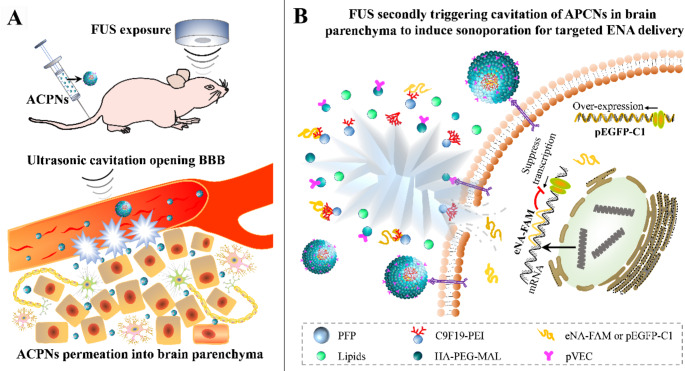
Two-step ultrasonic cavitation controlled delivery of brain exogenous nucleic acids for ischemic stroke using acoustic-cationic-polymeric-nanodroplets (ACPNs). A, first step ultrasonic cavitation (first FUS stimulated intravascular ACPNs cavitation) opened BBB to facilitate nonactivated ACPNs extravasation to ischemic brain parenchyma. B, second step ultrasonic cavitation (second FUS stimulated extravascular ACPNs cavitation) induced sonoporation for assisting the enter of exogenous nucleic acids to cells in ischemic parenchyma

**Supplementary Information:**

The online version contains supplementary material available at 10.1007/s13346-025-01828-6.

## Introduction

Stroke is one of the main causes of death and disability worldwide, and ischemic stroke (IS) accounts for approximately 85% of all strokes [1–3]. The main clinical IS treatment is the administration of thrombolytic agents such as recombinant plasminogen activator within 4.5 h of the acute onset time window or the removal of the embolus to achieve reperfusion. However, only approximately 5% of IS patients receive a timely treatment within the above time window [4, 5]. Moreover, most patients still suffer from brain dysfunction due to the damage of neurons and brain parenchyma even after an effective thrombolysis, showing movement and language disorders, even hemiplegia and loss of self-care ability [6–9]. Faced with such a dilemma, most investigators start to focus their attention to potential “chronic therapies” for IS. In this context, genetic therapies represent great potential to improve the recovery phase of brain ischemia [10]. Generally, the expression level of genes (mRNAs, miRNAs, circRNAs or lncRNAs, etc.) in brain cells after ischemia undergoes rapid changes to regulate pathophysiological processes, which represent potential therapeutic targets in the acute and subacute phase of IS [11–17]. The modulation of the aberrant genes allows alternative strategies using several approaches including miRNA mimics, antisense oligonucleotides (including siRNA and shRNA), gene overexpression vectors, and CRISPR [18, 19]. Typically, gene therapy requires the transfection of tens to thousands bp of exogenous nucleic acids (ENA) to correct abnormally expressed genes in brain ischemic cells.

Even more importantly, the efficacy of gene therapy on IS mainly depends on 2 crucial steps: ① opening the blood brain barrier (BBB) enables adequate extravasation of genetic materials or gene carriers into the ischemic brain parenchyma; ② targeted control of genetic material delivery to ischemic cells. Although viral systems have been recognized as an exquisite vehicle for gene transfection in scientific research and pre-clinical practice [20, 21], the high possibility of serious immuno-inflammation and carcinomatosis prevented their administration in clinical practice [22–24]. Non-viral vectors, including poly(ethylenimine) (PEI) [25], poly(amino esters) [26], poly(amino acid) [27], polyamide [28, 29], and chitosan [30] occupy important positions in the development of systems carrying genetic materials, but inefficient delivery efficiency and low precision limit their practical application [31]. Undoubtedly, the BBB primarily hinders the transfer of biopharmaceuticals (> 500 Da) from blood vessels to the brain parenchyma, where genetic materials and gene carriers function abnormally [32, 33]. IS or other brain diseases are usually lesions localized in the brain functional region, while the above viral or non-viral methods cannot specifically identify the blood vessels at the targeted lesion, often causing the wander of the genetic materials or gene carriers throughout the whole cerebrum. Hence, preferable strategies for opening the BBB should be investigated and implemented to effectively accumulate genetic materials or gene carriers into the ischemic brain parenchyma and to perform a targeted control of the genetic material delivery into ischemic cells to improve the effectiveness of genetic therapy on IS.

Previous studies applied focused ultrasound (FUS) plus artificial cavitation nuclei (i.e. microbubbles, nanobubbles, and nanodroplets) either to open BBB to control drugs release, or to induce sonoporation to enhance the uptake of substances by target cells [34–38]. Artificial cavitation nuclei are often used to reduce cavitation threshold avoiding the adverse reactions caused by high-intensity ultrasound. Particularly, the oscillation or collapse of microbubbles can interact with the endothelium of the brain vasculature and disrupt the tight junctions, thereby locally, transiently and reversibly increasing the permeability of BBB [32, 39, 40], showing tremendous potential for targeted extravasation and accumulation of carriers loaded with several factors including chemotherapeutic agents [41], antibodies [42, 43], genes [44, 45], or even cells [46]. The oscillations and even collapse of the microbubbles can also induce the transient formation of interstice on cell membrane, i.e., sonoporation, to facilitate exogenous substances delivery into targeted cells [47–49]. The sonication process has the advantages of being adjustable, noninvasive, and cost-effective [50]. However, microbubbles with an undesirably large size are restricted in the intravascular space, and cannot extravasate into the brain parenchyma through the BBB with limited opening scale [51]. Additionally, microbubbles and nanobubbles have a short circulation half-life of seconds to minutes, which is not enough to reach the target lesion [52, 53]. Fortunately, nanodroplets, a smart delivery platform with a smaller size and longer circulation half-life that vaporize into gas bubbles and undergo cavitation through FUS irradiation, can fulfill specific biophysical purposes [54–56]. Lea-Banks H et al. [54, 56] demonstrated that ultrasound-triggered nanodroplets effectively open the BBB and precisely perform drug delivery or neuromodulation in the brain to enhance theragnostic capabilities. Zhang et al. [51] developed PEGylated PLGA-based phase shift nanodroplets for BBB opening under FUS exposure, and confirmed that nanodroplets enhancing ultrasonic cavitation improve the precise distribution of Evan’s Blue (EB) within a narrow region in the center of a focal zone as compared with microbubbles. The residual nonactivated nanodroplets with a diameter ≤ 200 nm have a higher probability to extravasate through the opened BBB and permeate into the brain parenchyma, as demonstrated by Shen et al. [57], who revealed that liposomes with a diameter of 55 nm, 120 nm and 200 nm successfully pass through the opened BBB by FUS-targeted microbubble destruction. Additionally, the cell membrane is the second barrier for gene delivery, and again, sonication is the key solution to stimulate the phase transition and cavitation of extravascular nanodroplets to induce sonoporation for assisting the enter of genetic materials. The sonoporation requires that the cavitation nuclei are close enough to the cells, and the nanodroplets can extravasate more deeply and evenly spread around the ischemic cells. Although Chen et al. [54] used nanodroplets to deliver dextran within the targeted hippocampus during FUS irradiation, merely once sonication is limited to opening the BBB, but cannot stimulate the phase transition and cavitation of extravascular dextran-loaded nanodroplets to control dextran delivery into cells, because nonactivated dextran-loaded nanodroplets need sufficient time to extravasate across the opened BBB. Moreover, the process and mechanism of dextran delivery mediated by ultrasonic cavitation have not been fully elucidated. Although the research on intratumoral gene transfection controlled by ultrasonic cavitation was previously conducted by our group [47–49], transcranial ENA delivery poses greater challenges and its feasibility remains unknown. In summary, this work proposed a two-step ultrasonic cavitation scheme to address the above-mentioned two-step dilemma for brain gene delivery, in which the first ultrasound irradiation stimulated the intravascular nanodroplet cavitation for a reversible BBB opening to facilitate nonactivated nanodroplet extravasation, and the second ultrasound exposure induced extravascular nanodroplet cavitation in the brain parenchyma to control the targeted delivery of genetic materials, which deserves our attention and in-depth exploration effect and mechanism.

**Fig. 1:**
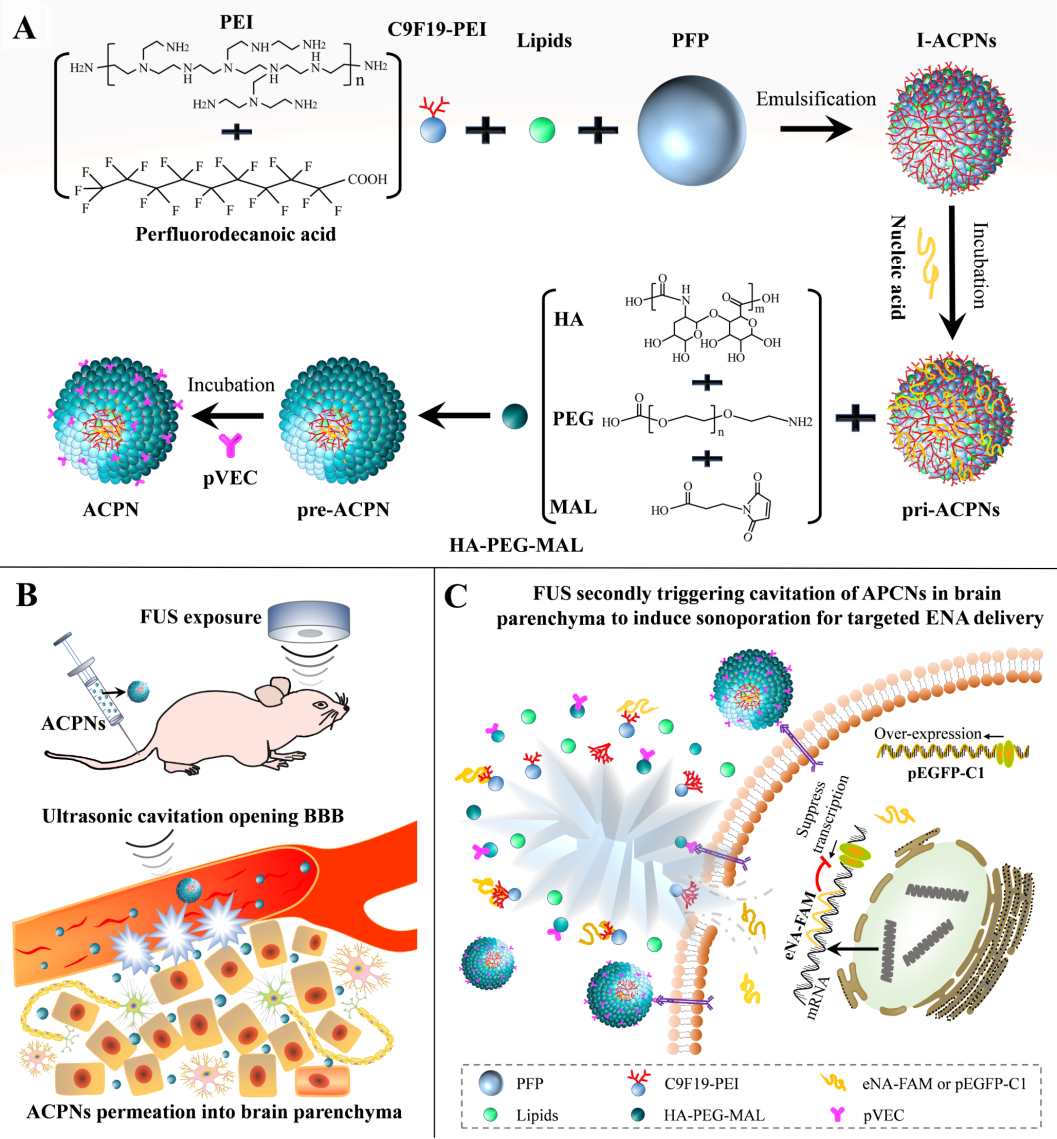
Analytical diagram of the ACPN structure during their preparation process and schematic graphic of the two-step ultrasonic cavitation to open BBB and to control ENA delivery respectively. **A** structural resolution during ACPN preparation process. **B** diagrammatic drawing of in vivo BBB opening through the first FUS stimulating intravascular ACPNs cavitation to facilitate nonactivated ACPNs extravasation to ischemic brain parenchyma across the opened BBB. **C** sketch map of the second FUS stimulating ACPNs cavitation in parenchyma to control ENA delivery to ischemic cells through sonoporation

Acoustic-cationic-polymeric-nanodroplets (ACPNs) were developed to efficiently carry ENA and protect them from enzymolysis, which contained the cationic amphiphilic fluorinated polymer C11F19-poly(ethylenimine) (C11F19-PEI) that not only efficiently encapsulated the perfluoropentane due to the hydrophobic perfluorinated segment (C11F19-), but they can also supply a hydrophilic cationic segment (-PEI) for ENA binding. The polymer hyaluronic acid-poly(ethylene glycol)-maleimide (HA-PEG-MAL) was selected to modify the surface of ACPNs to enhance the biocompatibility and the stability in serum, and to further conjugate the pVEC cell-penetrating peptides through the -MAL. Transient middle cerebral artery occlusion (tMCAO) rat model was subjected to sonication twice after the intravenous injection of ACPNs into the tail: firstly, FUS stimulated the phase transition and cavitation of intravascular large-sized ACPNs at a low intensity to open the BBB, and an interval of 2 h was set to allow a sufficient extravasation of the nonactivated ACPNs into the ischemic brain parenchyma (Fig. 1B). Subsequently, a second FUS stimulated the phase transition and cavitation of the oozed ACPNs in the brain parenchyma, where the ACPN phase-shifted microbubbles oscillated and collapsed to induce the sonoporation that controlled ENA delivery targeted to ischemic cells (Fig. 1C). This study not only demonstrated the application feasibility and mechanism of the two-step ultrasonic cavitation to control ENA delivery in IS lesion, but it might open up new avenues for the control of chemotherapeutic agent delivery, enhancement of stem cell homing, and modulation of cellular biophysical effects for the therapy of other brain neurological disorders.

## Materials and methods

### ACPNs preparation and characterization

#### Preparation of ACPNs

The ACPNs were formulated through ultrasonic emulsification, and the preparation process mainly included the following 5 steps, as shown in Fig. 1A.

Step 1: the perfluorodecanoic acid C11F19-COOH (Aladdin, Shanghai, China) and PEI (Aladdin, Shanghai, China) were dehydrated and condensed at a molar ratio of 1:1 to form C11F19-PEI. Afterwards, C11F19-PEI was dissolved in the precooled deionized water with a polymer concentration of 5 mg/mL. The 1 H NMR spectrum of C11F19-PEI dissolved in methanol is shown in Supplementary Fig. 1A. Matrix-Assisted Laser Desorption/Ionization Time of Flight Mass Spectrometry (MALDI-TOF MS) data showed a major ~ 8200 Da peak surround by several lower peaks (Supplementary Fig. 1B).

Step 2: the chloroform solution of C11F19-PEI, 1,2-dipalmitoyl-snglycero-3-phosphocholine, 1,2-dipalmitoyl-sn-glycero-3-phosphate, 1,2-distearoyl-sn-glycero-3-phosphoethanolamine-N-[methoxy(polyethylene glycol)-2000], 1,2-distearoyl-sn-glycero-3-phosphoethanolamine-N-[amino(polyethylene glycol)-2000] and cholesterol, purchased from Avanti Polar Lipids Inc. (Avanti, Alabaster, USA), was evaporated to form a thin film at a molar ratio of 7:1:1:1:2 in a rotary evaporator (Precision HLG3, Heidolph, Schwabach, Germany) under a nitrogen atmosphere, and then, the lipid film was hydrated with sterilized deionized water to obtain a 1 mg/mL suspension.

Step 3: 100 µL perfluoropentane (C_5_F_12_; Alfa, Zhengzhou, China) were quickly added to 5 mL lipid suspension, and sonicated using a sonicator (VX750, SONICS & MATERIALS, Inc., Newtown, USA) at 20% amplitude for 120 s (4 s on and 6 s off) in an ice bath to obtain the initial ACPNs (I-ACPNs). The redundant phospholipids, larger and smaller I-ACPNs were removed by centrifugation at 2000 rpm and 6000 rpm respectively to obtain the particles with a diameter of approximately 200 nm.

Step 4: chemosynthetic fragments of 30 bp of exogenous nucleic acids labeled with FAM fluorescein (named FAM-eNA) or pEGFP-C1 plasmids (4731 bp; expressing green fluorescent protein (GFP) in cells as a label for controlled pEGFP-C1 delivery) were mixed with I-ACPNs at a 1/2 P/N ratio, and further incubated at 4 °C for 30 min under stirring at 50 rpm to form the primary ACPNs (pri-ACPNs) by stepwise electrostatic interaction. Subsequently, the suspended nucleic acids were removed by centrifugation at 6000 rpm.

Step 5: hyaluronic acid (HA; Sigma-Aldrich, Saint Louis, USA), NH2-PEG2k-NH2 (Alfa, Zhengzhou, China), 6-maleimidocaproic acid-NHS (Aladdin, Shanghai, China) and triethylamine (Sigma-Aldrich, Saint Louis, USA) were dehydrated and polymerized at a molar ratio of 1:2:6:6 to form the polymeride HA-PEG-MAL. The 1 H NMR spectrum of HA-PEG-MAL dissolved in water is shown in Supplementary Fig. 1C. And MALDI-TOF MS of HA-PEG-MAL presented a peak MW being ~ 12,700 Da (Supplementary Fig. 1D). Next, superfluous HA-PEG-MAL was gently added to pri-ACPNs solution and incubated for 30 min to form the pre-ACPNs. Then pre-ACPNs were collected by centrifugation and mixed with the cell-penetrating peptide pVEC (Apeptide, Shanghai, China) followed by 30 min of incubation at 4 °C under stirring at 50 rpm to synthesize the ACPNs (i.e., FAM-eNA-ACPNs and pEGFP-C1-ACPNs), where -MAL conjugated with pVEC, and -HA integrated with dissociative -NH2 or -PEI through electrostatic interaction. The precipitate was collected by centrifugation at 6000 rpm and resuspended with deionized water for subsequent experiments (approximately 1 × 10^12^ ACPNs/mL).

#### Morphological characterization and stability analysis of ACPNs

The size distribution and zeta potential of I-ACPNs, pri-ACPNs and ACPNs were measured using the Malvern Nano Zetasizer (Malvern Instruments Ltd., Malvern, Worcestershire, U.K.). The morphology of ACPNs was observed by laser confocal fluorescence microscopy (LCFM; LEXT OLS4000, Olympus, Tokyo, Japan) and cryo-transmission electron microscope (c-TEM; Krios G4 Cryo-TEM, Thermo Fisher Scientific, Massachusetts, USA).

I-ACPNs at the concentrations of 0, 5, 10, 20, and 30 µL were mixed with 20 µL FAM-eNA (129.6 ng/µL) or pEGFP-C1 (159.87 ng/µL) to further evaluate the properties of ACPNs loading ENA. The supernatant and precipitate were collected by centrifugation at 8000 rpm after incubation at 4 °C for 30 min under stirring at 50 rpm, and the concentration was measured by a NanoDrop One microvolume UV-cis spectrophotometer (Thermo Fisher Scientific, Shanghai, China) and agarose gel electrophoresis was performed. Furthermore, the pri-ACPNs, ACPNs, FAM-eNA and pEGFP-C1 as the control were mixed with 20% serum at the same volume and subjected to agarose gel electrophoresis for the examination of ENA integrity.

In addition, the ACPNs were incubated in 37 ℃ water baths for 0–12 h, and the diameter was measured at one-hour intervals to monitor the stability of ACPNs.

#### Toxicity assays

The detection of the in vitro toxicity was performed by diluting the initial ACPN solution 2–20 times with fresh Dulbecco’s Modified Eagle Medium/Nutrient Mixture F-12 (DMEM/F-12; Gibco, Invitrogen, Carlsbag, USA), and the mixture was added to each well of 48-well plates (200 µL/well) containing 5 × 10^4^ neuroglial cells after removing the original medium. Cell plates were routinely placed into the incubator for 0–48 h, and cell viability was assessed by cell counting kit 8 (CCK-8; Absin, Shanghai, China).

Three-month male Sprague Dawley (SD) rats weighing from 200 g to 250 g, purchased from the Beijing Animal Experiment Center (Chinese Academy of Sciences, Beijing, China), were randomly divided into 2 groups: G1, the saline injected group; and G2, the ACPN injected group. Serum biochemical analysis was performed for the evaluation of the in vivo toxicity after intravenous injection of 0.5 mL normal saline or ACPNs for 5 days (once per day). Then the whole blood was harvested from the tail vein after rat anesthesia with isoflurane (RWD, Shenzhen, China), and centrifuged at 8000 rpm for 10 min at room temperature for 3 h. Then serum was collected from the supernatants and examined on a biochemical autoanalyzer to detect the content of creatine kinase (CK), alanine aminotransferase (ALT), aspartate aminotransferase (AST), total bilirubin (TBIL), creatinine (CREA), uric acid (UA) and blood urea nitrogen (BUN). The CK level indicated heart function. The liver function was evaluated by the assessment of the level of ALT, AST and TBIL, while kidney function was evaluated by the assessment of the content of CREA, UA, and BUN. In addition, the heart, liver, spleen, lung, and kidney were harvested, routinely fixed in paraffin wax, and subjected to pathological observation after staining with hematoxylin-eosin (HE) and TdT-dependent dUTP-biotin nick end labeling (TUNEL). All animal experiments were performed in strict accordance with the institutional guidelines, and approved by the Animal Experimentation Ethics Committee of Xi’an Jiaotong University (NO. XJTU_SLST2021-13).

### Acoustic field calibration

**Fig. 2:**
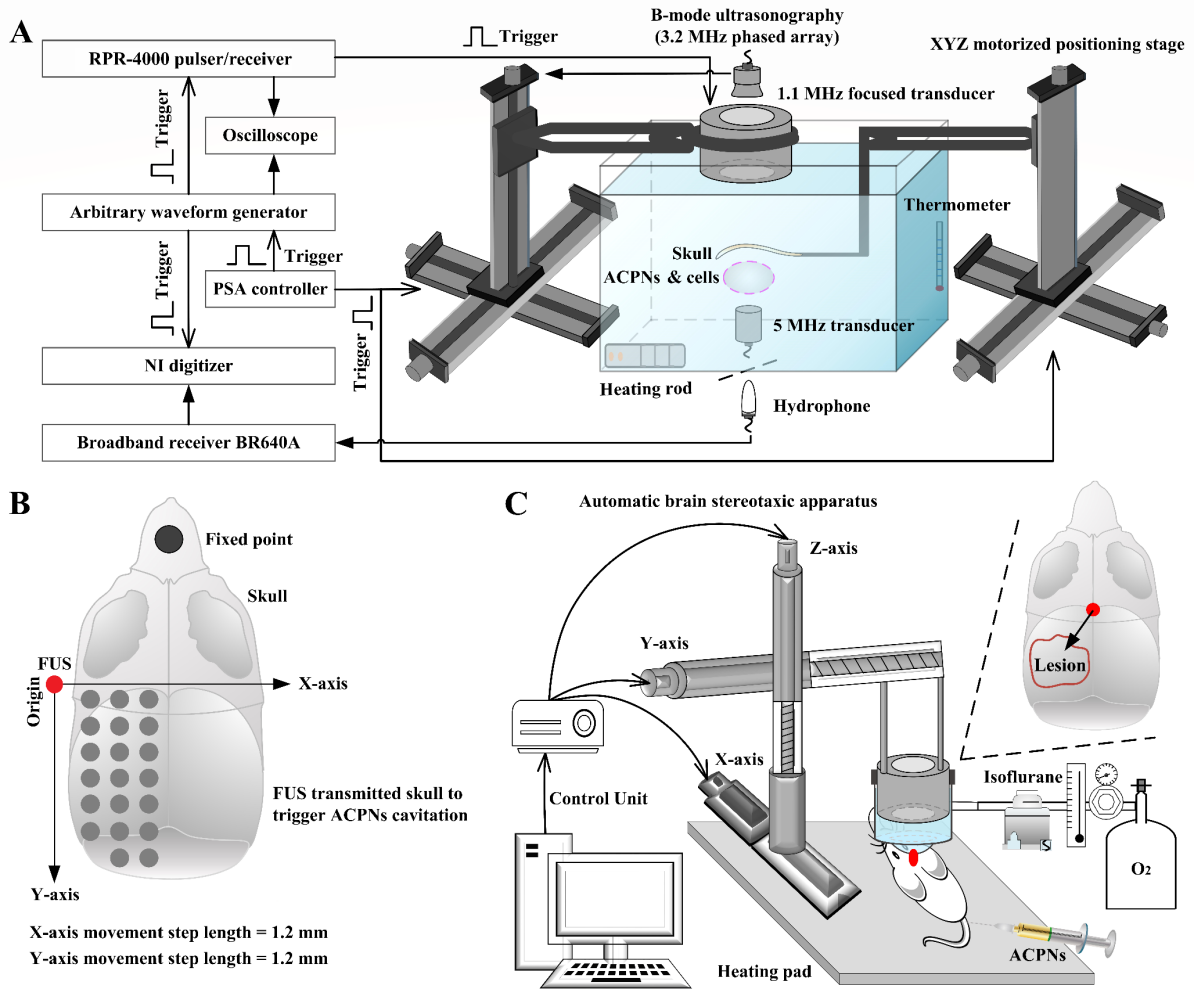
Schematic diagram of the experimental platform for in vitro and in vivo experiments. **A** experimental setup used for acoustic field calibration, transcranial FUS stimulating ACPNs cavitation, and ultrasonic cavitation impact on neuroglial cells viability in 3D hydrogel. **B** motion scheme of transcranial FUS irradiation displaced by a computer-controlled XYZ motorized positioning stage. **C** experimental platform of transcranial FUS stimulating ACPNs cavitation for opening BBB and controlling ENA delivery in vivo, where the automatic brain stereotaxic apparatus replaced the XYZ motorized positioning stage

The experimental setup for acoustic field characterization is shown in Fig. 2A. A 100-mm diameter 1.1 MHz spherically-focused transducer was driven through a pulser/receiver (RPR-4000, RITEC Inc., USA) that was triggered by the arbitrary waveform generator (33522B, Keysight, Beijing, China). A needle hydrophone (HNR-1000, ONDA, USA) was connected to a digitizer (5122, National Instruments Corp., Austin, TX, USA) to calibrate the acoustic pressure and spatial beam profile of FUS focal region. After positioning the needle hydrophone at the focus of the 1.1 MHz focused transducer, one computer-controlled XYZ motorized positioning stage (Zplix, Beijing, China) controlled the spatial motion of the 1.1 MHz transducer. The NI digitizer recorded the ultrasonic signals to calibrate the sound field. The FUS focal region was directly marked on the B-mode ultrasound image by the ultrasonic field detection to facilitate the process of focusing operation during the subsequent in vitro and in vivo experiments.

The transcranial sound field characterization was performed placing the rat skull above the hydrophone, which was displaced by another computer-controlled XYZ motorized positioning stage for the digitizer recording of ultrasonic signals after FUS penetration through different sites of the skull (Fig. 2B). The acoustic attenuation due to the presence of the skull was incorporated in the calibration in the water tank.

### Ultrasonic cavitation controlled ENA delivery in vitro

#### 3D hydrogel preparation

Chitosan (deacetylation 95% v/v, Macklin, Shanghai, China) solution (5% v/v hydrochloric acid solution) was mixed with β-glycerophosphate solution (50% w/w aqueous solution, Aladdin, Shanghai, China) at a volume ratio of 5:1. Then 1 mL rat tail type I collagen solution 0.1 g/mL (Beyotime, Shanghai, China) was diluted with 100 mL D-Hanks solution 10× (Solarbio, Beijing, China) containing NaOH at a concentration of 0.03 mmol/L. Furthermore, the two intermixtures mentioned above were mixed at the same volume ratio, and placed in an ice bath to obtain the 3D hydrogel solution. The structure of the solid hydrogel was observed by scanning electron microscopy (SEM; GeminiSEM-500, ZEISS, Oberkochen, Germany) after vacuum freeze-drying.

#### Transcranial FUS stimulated ACPNs cavitation

The 3D hydrogel mixed with ACPNs (approximately 5 × 10^7^ ACPNs/mL) was sealed in a 48-well cell culture plate with excellent acoustically transparent Parafilm M Laboratory Film (Bemis, Neenah, USA) after forming a gel in an incubator at 37 ℃, and immersed in degassed water maintained at 37 ℃. As shown in Fig. 2A, the cell culture plate was placed to the skull underface, ensuring that the FUS focal region was positioned in the plate hole under the B-mode image guidance. The arbitrary waveform generator triggering the RPR-4000 pulser/receiver generated a sinusoidal signal to drive the 1.1 MHz transducer emitting FUS with 0-5.4 MPa peak negative pressure (PNP). A 5 MHz single element focused transducer (309, Olympus, Japan) with a -6 dB bandwidth of 52.5% (2.15–6.9 MHz) connected to the digitizer was used to passively acquire echo signals for passive cavitation detection (PCD) during FUS irradiation on different sites of the skull (Fig. 2B). In addition, the 1.1 MHz and 5 MHz transducers were confocally fixed on the XYZ motorized positioning stage.

The passively received echo signals during FUS irradiation were processed using MATLAB (MathWorks, Natick, USA) for PCD analysis. The recorded waveforms were converted to frequency domain using fast Fourier transform. The stable cavitation dose (SCD) associated with stable nonlinear oscillation of the acoustically activated ACPNs was quantified based on the spectra around each subharmonic (*f*/2) and ultraharmonic (*nf*/2, *n* = 3, 5, 7, 9) frequency of each pulse. The inertial cavitation dose (ICD) associated with the inertial collapse of the microbubbles was quantified based on the broadband noise after filtering the fundamental (*f*), harmonic (*nf*, *n* = 1, 2, 3, 4), subharmonic and ultraharmonic signals. The SCD and ICD were calculated as the integrated area under the power curve of the time-cavitation noise over the entire duration of the recording. The ACPN phase transition and cavitation threshold were determined according to the sharp increase points of SCD and ICD with the continuous change of the ultrasound intensity. At least three data sets (each data set comprising 500 signals) per one experimental case were used to calculate the mean value of the SCD and ICD.

#### Ultrasonic cavitation impacted on cell viability

The neuroglial cells and ACPN solution were mixed with the prepared 3D hydrogel solution until reaching a density of approximately 5 × 10^6^ cells/mL and 5 × 10^7^ ACPNs/mL. The mixture was added into each well of 48-well plates, and allowed to form gel in an incubator at 37 ℃, saturated humidity and 5% CO_2_ for 20 min. Next, sufficient DMEM/F-12 supplemented with 10% fetal bovine serum (Gibico, Invitrogen, Carlsbag, USA), 1% penicillin/streptomycin (Gibico, Invitrogen, Carlsbag, USA), moderate glial cell-derived neurotrophic factor (Cyagen, Suzhou, China) and macrophage colony stimulating factor (Invitrogen, Carlsbag, USA) was added into the cell culture plates.

Based on PCD analysis revealing ACPN phase transition and cavitation threshold in part 2.3.2, specific ultrasonic parameters were schemed to explore the correlation between ultrasonic cavitation and neuroglial cell viability. The experimental procedure referred to the above content of FUS stimulating ACPN cavitation in 3D hydrogel. Briefly, the focus region of the 1.1 MHz transducer was positioned inside the 3D hydrogel (containing ACPNs and cells) according to the B-mode ultrasound guiding or echo signals received by the 5 MHz transducer. Whereafter, the 1.1 MHz transducer was moved across the whole 3D hydrogel through the XYZ motorized positioning stage (Fig. 2B). The digitizer recorded acoustic signals that were detected by the 5 MHz transducer for PCD analysis during FUS irradiation. After FUS treatment, the solidified 3D hydrogel was immersed into the mixture of lysozyme and collagenase in PBS (10 mM/L; pH = 7.4), and then incubated at 37 °C under shaking at 50 rpm. After the hydrogel changed from solid to liquid, the neuroglial cells were collected by centrifugation and subjected to the cell counting kit-8 (CCK-8; Engreen Biosystem, Beijing, China) assay. Subsequently, the neuroglial cell viability and ICD/SCD data were subjected to correlation analysis to reveal their relativity.

#### Ultrasonic cavitation controlled ENA delivery in vitro

The experimental FUS procedures for the controlled delivery of ENA in vitro are described in paragraph 2.3.3. After sonication, the fluorescent neuroglial cells in solid hydrogels were observed by the LCFM. Then the neuroglial cells were separated from the 3D hydrogel, and analyzed by flow cytometry (CytoFLEX LX, Beckman Coulter Life Sciences, Indiana, USA) to calculate the ratio of FAM or GFP fluorescent cells. Specifically, FAM fluorescent cells were detected within 60 min after FUS irradiation (Supplementary Fig. 2A), while pEGFP-C1-delivered cells were cultured for 48 h before GFP fluorescence detection (Supplementary Fig. 2B).

### Two-step ultrasonic cavitation controlled ENA delivery in vivo

#### Preparation of tMCAO models

Three-month male SD rats weighing from 200 g to 250 g were subjected to transient middle cerebral artery occlusion (tMCAO) to obtain the IS model. During the experiment, the rats were fed with sterilized food and water ad libitum and housed in IVC independent cages under a 12 h light*/*dark cycle, controlled temperature at 25 °C and 30–50% humidity. After anesthesia with isoflurane, the rats were subjected to permanent focal cerebral ischemia by ligature of the left common carotid artery and occlusion of the ipsilateral distal middle cerebral artery using a silicon-coated 6 − 0 nylon filament, and the embolus was withdrawn after 70 min reperfusion.

The cerebral ischemic territory was measured using 2,3,5-triphenyl-2 h-tetrazolium chloride (TTC) staining. Briefly, the rats were deeply anesthetized with isoflurane, and cardiac perfusion was performed using 200 mL normal saline. Then, the brain was quickly removed and cut into 2 mm-thick sections on the coronal plane. Each section was immersed in 1% TTC at 37 °C for 30 min for vital staining.

#### First ultrasonic cavitation opened BBB to enhance ACPNs extravasation

The experimental setup shown in Fig. 2C, was similar to that of in vitro ENA delivery experiment except that the rat’s head was coupled to the ultrasound transducer by a coupling cone containing degassed water, and particularly using the automatic brain stereotaxic apparatus to carry the 1.1 MHz transducer for guiding FUS to accurately irradiate specific brain locations. The rat hairs on the scalp were removed with an electric clipper and depilatory creams before subsequent experiments. After the intravenous injection of 0.5 mL ACPNs for 0.5 h, the rat was placed on a heated pad to maintain the body temperature, and the anesthesia was maintained by delivering isoflurane in 100% oxygen. Then, the degassed coupling agents were applied on the scalp, and the Z-axis stage was adjusted to allow the head to come into close contact with the acoustic window on the cone bottom. Next, the automatic brain stereotaxic apparatus was digitally manipulated to move the focus of 1.1 MHz transducer into the interior of the left hemisphere.

BBB opening by first ultrasonic cavitation was verified by the qualitative and quantitative assessment of EB (Aladdin, Shanghai, China) extravasation. After FUS treatment, the tMCAO rats were treated with an intravenous injection of EB (100 mg/kg) instantly, and further raised for 2 h. Afterwards, the rats were sacrificed, and their brains were removed for direct observation. The right and left hemispheres were separated on the midline, weighed and soaked in 50% trichloroacetic acid solution. After homogenization and centrifugation, the extracted EB dye was diluted in absolute ethyl alcohol, and the optical density was measured by a Thermomax microplate reader (Bio-Tek, Winooski, USA) at 620 nm. The EB content among each hemisphere was quantified by a linear regression standard curve derived from five concentrations of the dye, and denoted in terms of the amount per gram of hemisphere tissue to determine the BBB opening magnitude [51].

Additionally, ACPNs labeled with rhodamine B (RB) were used to visualize the permeability of the ACPNs into the cerebral ischemic lesion through the opened BBB, which were prepared with RB-cholesterol instead of cholesterol. Following 2 h plastochrone after FUS exposure, the tMCAO rats were sacrificed by an overdose of the anesthetic isoflurane. The brain was quickly extracted, and coronally sliced for 4 sections at equal intervals and subjected to RB fluorescence imaging by IVIS LUMINA living imaging system (ILLIS; Caliper Life Sciences, California, USA). Subsequently, the brain was embedded into an optimal cutting temperature compound for frozen section cutting, and then subjected to fluorescence observation using an Axioscan 7 slide scanning system (ZEISS, Oberkochen, Germany). The main experimental process is shown in Supplementary Fig. 2C. Moreover, all the experimental brains were embedded in paraffin and cut for hematoxylin and eosin (H&E) staining and transferase-mediated deoxyuridine triphosphate-biotin nick end labeling (TUNEL) to confirm the safety of the procedure.

#### Second ultrasonic cavitation for controlling IS-targeted ENA delivery

After the penetration of the nonactivated ACPNs into the cerebral ischemic lesion, the rats were subjected to FUS irradiation again to perform the targeted intracellular ENA delivery into the ischemic cells by the surrounding ACPNs accumulated in the lesion. The operation procedure of the ultrasound irradiation was performed exactly as described in Sect. “First ultrasonic cavitation opened BBB to enhance ACPNs extravasation”.

The detection of FAM-eNA delivery (Supplementary Fig. 2D) was performed by extracting quickly the brains at an interval of 0.5 h after second sonication, 4 sections were cut on the coronal plane at equal intervals along the longitudinal axis, and subjected to ILLIS imaging to monitor FAM fluorescence. The fluorescence intensity was calculated to elucidate the effect of ultrasonic cavitation controlling FAM-eNA delivery in the ischemic brain parenchyma. Soon afterwards, the brain was sectioned for FAM fluorescence observation by the LCFM, and the FAM fluorescence area ratio was calculated using the Image J (National Institutes of Health, Bethesda, USA). The number of FAM fluorescent cells was quantified after the brain was processed to form a single cell suspension using flow cytometry.

The tMCAO rats were fed for another 2 days after FUS irradiation to monitor the pEGFP-C1 plasmid delivery (Supplementary Fig. 2E). The tMCAO rats were sacrificed with an excess of isoflurane, and the brains were collected and coronally sliced for GFP fluorescence imaging by ILLIS, and further cut after being frozen for GFP fluorescence monitoring by the LCFM. Moreover, flow cytometry was emphatically utilized to quantify GFP fluorescent cells.

### Statistical analysis

Statistical analyses were conducted using GraphPad Software (version 8.0.1; GraphPad Software Inc., La Jolla, California, USA). Two-group comparisons were made using a two‐tailed Student’s t‐test assuming unequal variances. Regarding to multiple‐group (> 2 groups) comparisons, one‐way analysis of variance (ANOVA) with Bonferroni’ multiple comparisons test was employed. Results were expressed as average ± standard error of mean (SEM) from at least three independent experiments. Differences were determined to be statistically significant at *p* < 0.05 (e.g., *, *p* < 0.05; **, *p* < 0.01; and ***, *p* < 0.001).

## Result and discussion

### ACPNs characterization

Transient pores on cell membrane offered an ephemeral duration for the intracellular transfer of genetic materials [36], and previous studies demonstrated that the plasmid delivery efficiency is much higher in the microbubble-plasmid complexes than in the mixture of microbubbles and plasmids alone [58, 59]. Therefore, this study constructed the ACPNs, a holistic system, with ENA adsorbed onto the surface by electrostatic performance.

**Fig. 3:**
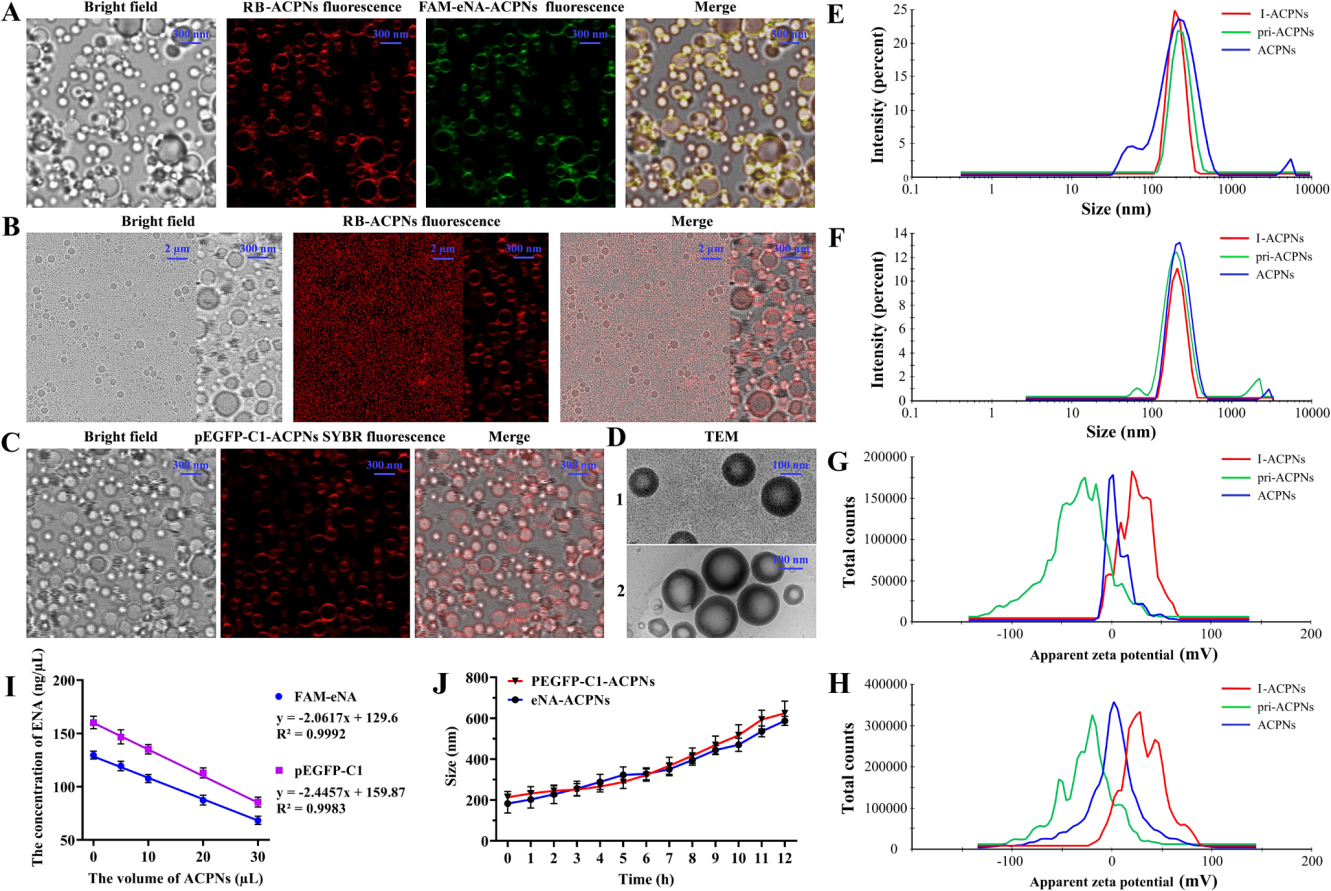
Morphological characterization and stability analysis of ACPNs. **A** LCFM images of FAM-eNA-ACPNs, including bright field image of ACPNs, image of RB-cholesterol or FAM-eNA within ACPNs, and the merged image. **B** LCFM images of pEGFP-C1-ACPNs, encompassing bright field imaging of ACPNs, imaging of RB-cholesterol, and the merged image. **C** LCFM images of pEGFP-C1-ACPNs after pEGFP-C1 labeled with SYBR. **D** c-TEM images of ACPNs (1, FAM-eNA-ACPNs; 2, pEGFP-C1-ACPNs). **E-F** size distribution of I-ACPNs, pri-ACPNs, and ACPNs during the preparation of FAM-eNA-ACPNs or pEGFP-C1-ACPNs respectively. **G-H** zeta potential of I-ACPNs, pri-ACPNs, and ACPNs during the preparation of FAM-eNA-ACPNs or pEGFP-C1-ACPNs respectively. **I** ENA concentration detection by a NanoDrop One microvolume UV-cis spectrophotometer to calculate the loading levels of FAM-eNA or pEGFP-C1 on ACPNs. **J** diameter changes of FAM-eNA-ACPNs and pEGFP-C1-ACPNs incubated at 37℃ for 12 h. Four replicates were designed for each experimental group

The results of LCFM and c-TEM in Fig. 3A-D showed that the ACPNs loading FAM-eNA (FAM-eNA-ACPNs) or pEGFP-C1 (pEGFP-C1-ACPNs) were spherical with a mean diameter of 200 nm. Additionally, the mean diameter of I-ACPNs, pri-ACPNs, and ACPNs, detected by the Malvern Nano Zetasizer, did not significantly change, and fluctuated around 200 nm (Fig. 3E-F). The size of ACPNs was comparable to the size of materials reported by Shen et al. [57] that can pass the opened BBB induced by ultrasonic cavitation. According to Malvern Nano Zetasizer test results shown in Fig. 3E-F, large-size ACPNs even up to 400 nm were also present, which was in accordance with our expectation that the low-intensity FUS initially stimulated ACPN phase transition and cavitation to open BBB (Fig. 1A). Thus, nonactivated ACPNs are promoted to exosmose through the opened BBB into the ischemic brain parenchyma (Fig. 1B).

LCFM fluorescence images showed that FAM-eNA and pEGFP-C1 were effectively adsorbed and evenly distributed on the outer layer of the ACPNs (Fig. 3A-C). In the process of ACPN preparation, the I-ACPNs possessed a significant positive potential of 13.4 ± 3.2 mV; after adsorbing ENA, the pri-ACPNs showed a significant negative potential of -21.6 ± 2.3 mV; and the final ACPNs showed a potential of approximately 0 mV (Fig. 3G-H). The potential changes of the intermediates during ACPN preparation proved that ENA was loaded onto the ACPNs, which was consistent with our previous report demonstrating that the positive potential -PEI or -NH2 adsorbs the negative potential ENA [47–49].

In addition, the results of ENA concentration detected by a NanoDrop One microvolume UV-cis spectrophotometer and agarose gel electrophoresis proved that ACPNs effectively carried FAM-eNA or pEGFP-C1 (Fig. 3I; Supplementary Fig. 3). The loading efficiency of FAM-eNA on ACPNs was 41.234 ng/µL (Fig. 3I) calculated by the variation of ENA concentration relative to the amount of I-ACPNs, and the amount of pEGFP-C1 loaded on ACPNs was 48.914 ng/µL (Fig. 3J). Strikingly, gene therapy is greatly weakened due to the rapid clearance of nucleic acids in the bloodstream by enzymatic degradation [47]. The results of agarose gel electrophoresis further confirmed that ACPNs effectively protected ENA from decomposition by nucleases in serum (Supplementary Fig. 3), which ensured the feasibility of their in vivo application. Additionally, RB-labled pEGFP-C1-ACPNs, as a representative case, were prepared with FITC-pVEC instead of pVEC to evaluate the effect of pre-ACPNs coupling pVEC. LCFM fluorescence images showed a significant FITC fluorescence at the pEGFP-C1-ACPNs edges (Supplementary Fig. 4A), and -MAL conjugated with pVEC at a concentration of 724.63 ± 36.28 ng/mL (Supplementary Fig. 4B).

The mean diameter of ACPNs at 37 °C was ≤ 230 nm within 3 h, and was ≤ 300 nm within 6 h, which fully satisfied the requirements of carrier stabilization within 3 h in subsequent experiments in vitro and in vivo (Fig. 3J). In addition, pEGFP-C1-ACPNs, as a representative, were subjected to toxicity safety tests in vitro and in vivo. The results of CCK-8 toxicity assay showed that the original pEGFP-C1-ACPNs diluted for 5–20 times did not exert any toxicity to cells in vitro, but 2-fold dilution of pEGFP-C1-ACPNs resulted in a significant decrease in cell viability (Table 1). According to our previous study [49], it is reasonable to believe that the decrease in neuroglial cell viability was not due to the toxicity of ACPNs, but the high concentration of ACPNs caused a prolonged gas obstruction in the cell culture system. Notably, the results of serum biochemical analysis showed that pEGFP-C1-ACPNs did not exert any toxicity to the body (Table 2).

**Table 1:** Relative cell viability of neuroglial cells after incubation with pEGFP-C1-ACPNs diluted for 2–20 times at 37℃, saturated humidity and 5% CO_2_ for 0–48 h. Five replicates were designed for each experimental group

Incubation time (h)	Dilution ratio of ACPNs (times)			
	2 (Average ± SEM)	5 (Average ± SEM)	10 (Average ± SEM)	20 (Average ± SEM)
0	1.075 ± 0.008	1.044 ± 0.007	1.016 ± 0.008	1.028 ± 0.011
12	0.864 ± 0.014 *	0.924 ± 0.011	0.983 ± 0.011	0.982 ± 0.007
24	0.772 ± 0.012 **	0.942 ± 0.015	0.961 ± 0.009	1.033 ± 0.013
48	0.817 ± 0.017 **	0.917 ± 0.012	0.972 ± 0.013	1.017 ± 0.009

**Table 2:** Serum biochemical analysis of pEGFP-C1-ACPN toxicity on heart, liver and kidney function. Liver function was evaluated from the levels of serum Alanine aminotransferase (ALT) and aspartate aminotransferase (AST), and the total bilirubin levels (TBIL). Kidney function was evaluated from the levels of serum creatinine (CREA), uric acid (UA) and blood Urea nitrogen (BUN). And heart function was evaluated from the level of serum creatine kinase (CK). G1, the saline injected group; G2, the ACPN injected group. Five replicates were designed for each experimental group

Biochemical analysis	G1 (Average ± SEM)	G2 (Average ± SEM)
ALT (U/L)	141.13 ± 8.00	128.56 ± 8.94
AST (U/L)	256.57 ± 7.03	265.07 ± 10.08
TBIL (µmol/L)	11.50 ± 0.37	11.64 ± 0.56
UREA (mg/dl)	24.99 ± 0.71	25.17 ± 0.62
CREA (µmol/L)	26.42 ± 0.58	23.39 ± 0.41
UA (µmol/L)	156.50 ± 6.48	139.48 ± 7.12
CK (U/L)	2984.89 ± 115.76	3002.93 ± 181.67

### Acoustic field calibration, transcranial FUS stimulated ACPNs cavitation, and ultrasonic cavitation controlled ENA delivery in vitro

#### Acoustic field calibration

**Fig. 4:**
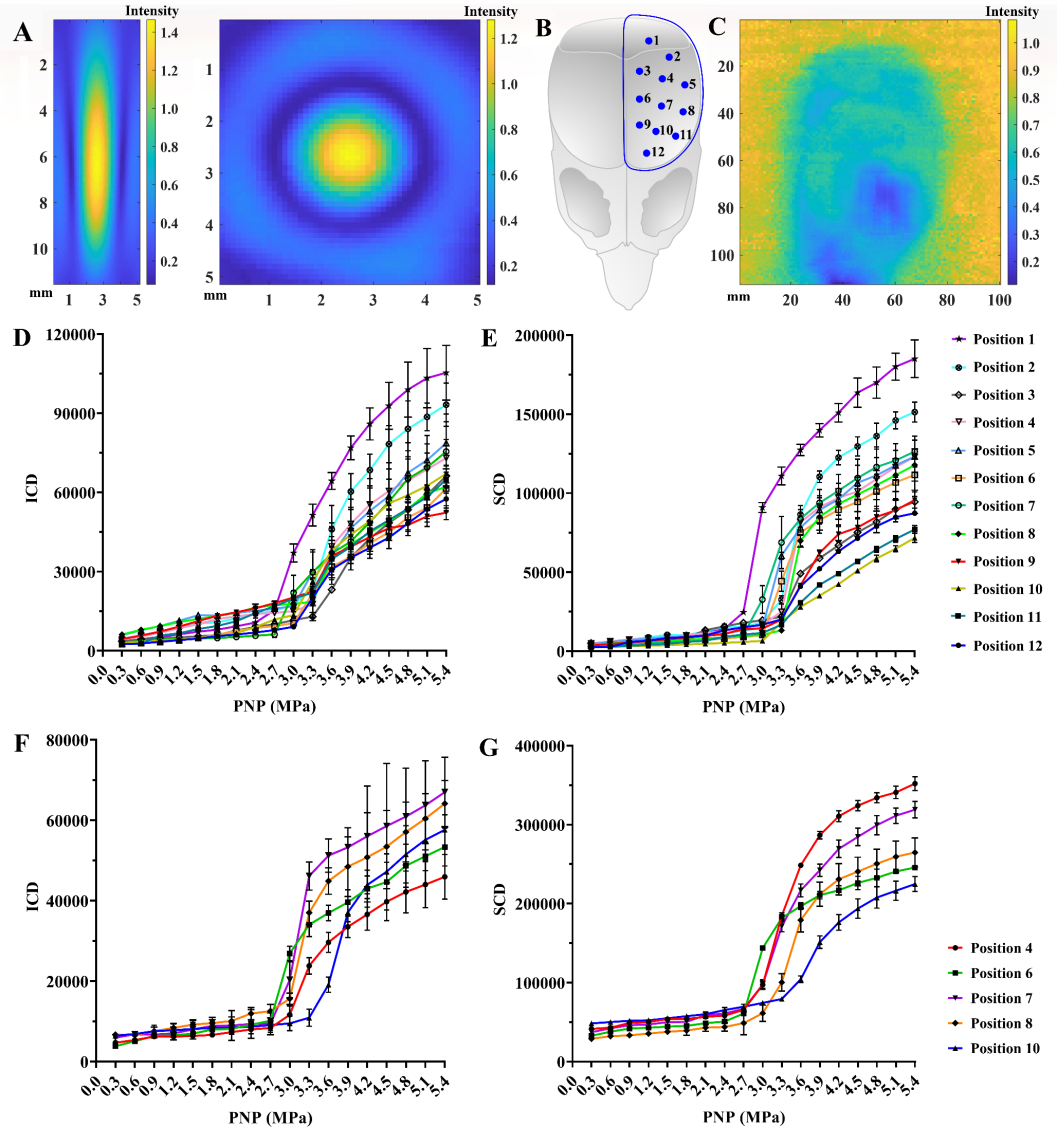
Longitudinal and horizontal spatial beam profile of FUS focal region, acoustic characteristics of transcranial FUS, and PCD analysis of FUS stimulating ACPNs. **A** longitudinal and horizontal spatial beam profile of FUS focal region at the point of maximum ultrasonic intensity respectively. **B** a schematic diagram for detecting transcranial FUS acoustic characteristics irradiating the left-brain skull that were highlighted in a blue semicircle, and selecting 12 sites on the skull to verify the feasibility of transcranial FUS stimulating ACPNs cavitation. **C** map of ultrasonic intensity after FUS penetrating through the full-scale of left-brain skull. **D-E** ICD and SCD of FAM-eNA-ACPNs excited by transcranial FUS irradiating 12 different left-brain skull sites under various PNP, respectively. **F-G** ICD and SCD of pEGFP-C1-ACPNs irradiated by FUS penetrating through 5 different left-brain skull positions under different PNP parameters. Four replicates were designed for each experimental group

As calibrated through the needle hydrophone and digitizer, the 1.1 MHz transducer had a spherical face with a 65-mm radius of curvature, of which the longitudinal and horizontal spatial beam profile of FUS focal region at the point of maximum ultrasonic intensity presented a regular rugby ball shape and regular round shape respectively, and the ultrasonic intensity gradiently decreased from the center to the edge (Fig. 4A). The pressure profile has a full width at half amplitude of 1.2 mm laterally and 6.5 mm in the depth direction as FUS emitted by the 1.1 MHz transducer at parameters of 1.2 MPa, 100 cycles and 20 Hz (Fig. 4A). According to the actual measurements, the pressure profile dimensions were closely related to the ultrasonic intensity. Additionally, the maximum negative pressure and the maximum positive pressure were nearly equal, and the pressure varied linearly with the driving voltage.

Subsequently, the ultrasonic intensity of FUS penetrating through the left-brain skull (highlighted in a blue semicircle in Fig. 4B) is shown in Fig. 4C, which directly revealed the FUS attenuation by the skull. In detail, FUS penetrated the skull (with an area rate surpassing 85%) with a reduction in intensity by approximately 35%, but it was significantly hampered near the orbital part of the skull with an obstruction rate exceeding 70% (Fig. 4C).

#### Transcranial FUS stimulated ACPNs cavitation

According to our previous reports [47–49], FUS stimulating the phase transition and cavitation of ACPNs in the brain parenchyma was a necessary condition for the controlled delivery of ENA. Moreover, PNP was the predominant factor for a successful phase transition and cavitation of nanodroplets compared to pulse duration (PD), pulse repetition frequency (PRF) and exposure time (ET) [47, 49, 60, 61]. Therefore, the feasibility of FUS in penetrating the skull (12 sites marked by blue dots in Fig. 4B) to stimulate ACPN cavitation was further verified under various PNP, 100 cycles, 20 Hz and 5 s as fixed parameters. The results of PCD analysis demonstrated that FUS effectively stimulated the phase transition-cavitation of FAM-eNA-ACPNs through 12 different sites of the skull (Figure D-E). Once exposed to sufficient rarefactional pressures, ACPNs were firstly phase shifted to microbubbles that started to oscillate in a similar manner as traditional microbubbles. The phase transition threshold of FAM-eNA-ACPNs, as indicated by the curves of ICD and SCD versus PNP, was 2.7–3.3 MPa, mainly using 3 MPa as a reference (Fig. 4D-E). It primarily depended on the ultrasound attenuation degree at different sites of FUS penetrating the skull (Fig. 4B-C). Subsequently, considering the negligible morphological difference between pEGFP-C1-ACPNs and FAM-eNA-ACPNs, our hypothesis was that no evident variation might appear in the phase transition threshold. As such, five sites on the skull were chosen to detect FUS-stimulated pEGFP-C1-ACPNs phase transition-cavitation outcomes. The PCD results confirmed our hypothesis that the phase transition threshold of pEGFP-C1-ACPNs was 2.7–3.3 MPa, also referred to as 3 MPa as the phase transition threshold (Fig. 4F-G). ICD and SCD were significantly high as PNP ≥ 3 MPa and increased, which meant that more and more ACPNs were phase shifted to bubbles that oscillate and collapse. Undoubtedly, PCD revealing 3 MPa phase transition threshold of ACPNs served as a crucial reference for the selection of FUS parameters in subsequent in vitro and in vivo experiment to open BBB and control ENA delivery.

**Fig. 5:**
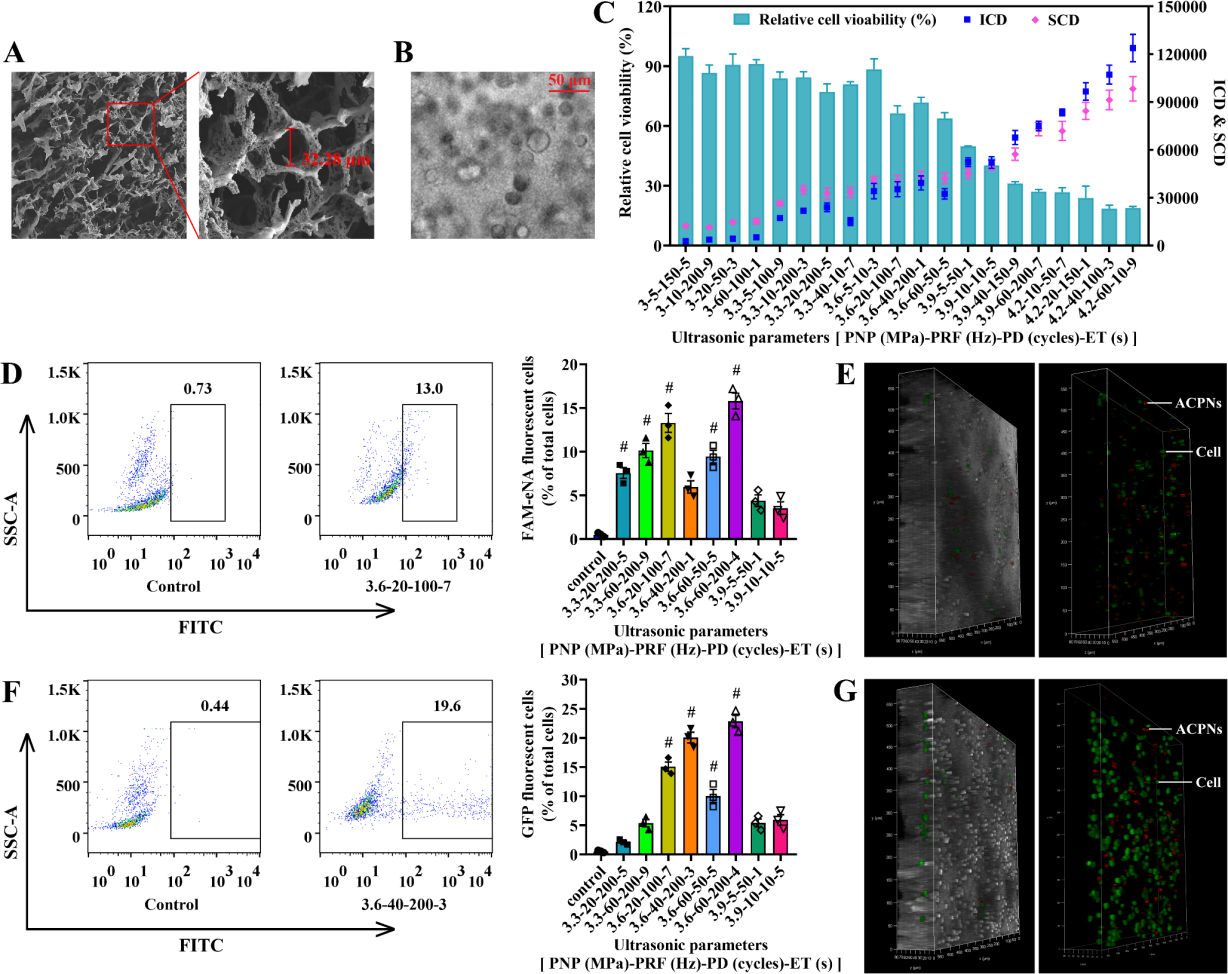
Ultrasonic cavitation controlled ENA delivery to neuroglial cells in 3D hydrogel. **A** SEM of the freeze-dried 3D hydrogel. **B** LCFM image of the cells cultured in 3D hydrogel. **C** correlation analysis of the neuroglial cells viability and ICD/SCD under different ultrasonic parameters. **D** flow cytometry of FAM fluorescent cells after transcranial FUS stimulating FAM-eNA-ACPNs cavitation to control FAM-eNA delivery under different ultrasonic arguments. **E** LCFM images of FAM fluorescent neuroglial cells after transcranial FUS stimulating FAM-eNA-ACPNs cavitation for FAM-eNA controlled delivery under 3.6 MPa, 20 Hz, 100 cycles, and 7 s parameters. **F** flow cytometry of GFP fluorescent neuroglial cells after FUS penetrating the skull to stimulate pEGFP-C1-ACPNs cavitation for controlling pEGFP-C1 delivery under different ultrasonic parameters. **G** LCFM images of GFP fluorescent neuroglial cells after transcranial FUS stimulating pEGFP-C1-ACPNs cavitation for pEGFP-C1 controlled release under 3.6 MPa, 40 Hz, 200 cycles, and 3 s parameters. The ultrasonic parameters in the figure are labeled as PNP-PRF-PD-ET, of which the unit is MPa-Hz-cycles-s. #, indicating that the data are significantly higher than the other treatment groups (*p* < 0.05), and significantly higher than the control group (*p* < 0.001). Three biological replicates were designed for each experimental group

It is noteworthy that the aforementioned experiments of transcranial FUS stimulating ACPN cavitation were performed in 3D hydrogel comprising ACPNs for a more realistic simulation of brain parenchyma. Figure 5A shows that the SEM images of freeze-dried 3D hydrogel presented an interior honeycomb structure with a diameter of 34.72 ± 4.64 μm, which satisfied the requirement of cell survival space. Importantly, neuroglial cells cultured in 3D hydrogel showed a normal morphology as shown in Fig. 5B.

Subsequently, according to the phase transition threshold of ACPNs stimulated by transcranial FUS (Fig. 4D-G), the correlation of ultrasonic cavitation and neuroglial cell viability was further evaluated in 3D hydrogel based on the orthogonal experiments of PNP, PD, PRF and ET with 5 levels, as listed in Table 3. The results of PCD and cell viability were jointly analyzed as shown in Fig. 5C. When PNP was set at 3 MPa, the ICD and SCD showed no significant changes as PD or PRF increased. However, when PNP increased to ≥ 3.3 MPa, the ICD and SCD were positively correlated with the PD or PRF. It fully demonstrated that PNP played a crucial role in ACPN phase transition and cavitation and adjusting PD or PRF after PNP higher than the phase transition threshold affected the fluctuation of the cavitation dose within a certain range. The cell viability significantly decreased with increasing ICD and SCD, which indicated a cavitation dose-dependent relationship with cell damage. The in-depth analysis revealed that the amplification of PNP led to the increase of the cavitation dose per unit time, resulting in the decrease of neuroglial cell viability. When the cavitation dose per unit time remained constant, prolonging the action time of FUS (by increasing PD, PRF, or ET) led to a greater cavitation dose that compromised cell viability. According to our previous studies, ICD had a more pronounced impact on cytoactivity compared to SCD [47–49]. However, the initial cavitation and stable cavitation of ACPN-phase-shifted microbubbles did not show any significant magnitude difference in dose statistics due to the skull obstruction to ultrasound and steric restraints of hydrogel pores; thus, the effect of ICD and SCD on cell viability was not clearly demonstrated.

**Table 3:** Four ultrasonic parameters and five levels orthogonal experiments design. The PD and PRF were regulated through the manual trigger arbitrary waveform generator, and PNP was adjusted by the pulser/receiver RPR-4000. And ET was modulated by the moving scheme of the XYZ stage, 1.1–0.12 mm/s moving speed corresponded to ET of 1–9 s

Factors	Parameter levels				
PNP (MPa)	3	3.3	3.6	3.9	4.2
PRF (Hz)	5	10	20	40	60
PD (Cycles)	10	50	100	150	200
ET (s)	1	3	5	7	9

#### Ultrasonic cavitation controlling ENA delivery in vitro

Based on the correlation between cell viability and cavitation dose (Fig. 5C), 8 ultrasonic parameters were chosen and designed for transcranial FUS safely and effectively stimulating ACPN cavitation to facilitate ENA controlled delivery into neuroglial cells in 3D hydrogel.

Regarding the controlled delivery of FAM-eNA, the flow cytometry results indicated an evident increase in the number of FAM fluorescent neuroglial cells in the 8 groups as compared to the control group (Fig. 5D). Within these groups, 3.3-20-200-5, 3.3-60-200-9, 3.6-20-100-7, 3.6-60-50-5, and 3.6-60-200-4 groups showed a significantly higher number of FAM fluorescent neuroglial cells compared to that in the remaining treatment groups (Fig. 5D). However, the outcomes of cavitation dose *versus* cell viability in Fig. 5C revealed that the parameters of 3.3-20-200-5, 3.3-60-200-9 and 3.6-20-100-7 were optimal in facilitating FAM-eNA controlled delivery to neuroglial cells. Additionally, LCFM images showed that the number or percentage of FAM fluorescent neuroglial cells *versus* the overall cells was relatively larger within the 3D hydrogel of the 8 groups after transcranial FUS irradiation at the parameters of 3.3-20-200-5, 3.3-60-200-9 and 3.6-20-100-7 (Fig. 5E).

As regards the delivery of pEGFP-C1, flow cytometry demonstrated that the number of GFP fluorescent neuroglial cells in seven of the eight treatment groups except the 3.3-20-200-5 group was significantly higher than that in the control group. In addition, the number of GFP fluorescent neuroglial cells was significantly higher in the 3.6-20-100-7, 3.6-40-200-3, 3.6-60-50-5, and 3.6-60-200-4 groups than the others. In light of the cavitation dose *versus* cell viability shown in Fig. 5C, the parameters of 3.6-20-100-7 and 3.6-40-200-3 were the most optimal for controlled pEGFP-C1 delivery in targeted brain lesions. Concurrently, LCFM images revealed that the quantity or proportion of GFP fluorescent neuroglial cells relative to total cells was relatively higher in 3D hydrogel after exposure to FUS under 3.6-20-100-7 and 3.6-40-200-3 ultrasonic parameters among the 8 groups (Fig. 5E).

Generally, the delivery of macromolecules into cells is more difficult than micromolecules. The ultrasonic parameters required for 5000 bp pEGFP-C1 delivery were 3.6-20-100-7 or 3.6-40-200-3, which were higher than those required for 50 bp FAM-eNA delivery at 3.3-20-200-5, 3.3-60-200-9 or 3.6-20-100-7. This means that the amplitude of cell membrane pores (sonoporation) induced by transcranial FUS stimulating pEGFP-C1-ACPNs cavitation was larger than that inducted by FUS stimulating FAM-eNA-ACPNs cavitation to meet the demand for larger molecular pEGFP-C1 delivered to cells. In terms of ultrasonic waveforms interpreted in stages according to Fig. 4D-G, ACPNs stimulated by FUS at 3 MPa were phase-shifted to bubbles that continued to oscillate, while the bubbles showed a larger oscillation amplitude and even collapsed at ≥ 3.3 MPa that resulted in feasible membrane holes for FAM-eNA delivery (Supplementary Fig. 5). However, much more bubbles oscillated with larger amplitude and collapsed at 3.6 MPa compared to 3.3 MPa, and higher mechanical force led to more prominent cell membrane pores for pEGFP-C1 delivery. From the perspective of cavitation dose, the condition of 3.6 MPa resulted in more small-sized ACPNs phase-changed into bubbles, in turn followed by larger oscillation and crumble as compared with the condition ≥ 3.3 MPa, which instantaneously generated larger mechanical effect eliciting larger size membrane holes, thereby promoting the entry of the macromolecule pEGFP-C1 into the cells (Supplementary Fig. 5). The tradeoff came as higher cavitation dose or longer sonication duration created more “collateral damage” to the nearby cell or tissue, while it provided an extravascular delivery in a greater extent [62].

### Two-step ultrasonic cavitation controlled ENA delivery in vivo

#### First ultrasonic cavitation opened BBB to enhance nonactivated ACPNs extravasation

Gene therapy is a promising strategy for cerebrovascular and neurological diseases [63], but the noninvasive delivery of therapeutical genetic materials is seriously hindered by the BBB as it prevents large molecules (> 500 Da) from extravasating into the brain parenchyma [32, 33]. Although the paragraph 3.2 described that ultrasonic cavitation successfully controlled ENA delivery in vitro 3D hydrogel to imitate the intracranial situation, the in vivo opening of the BBB to ensure extravasation of ACPNs to the parenchymal lesions of the ischemic brain was the crucial prerequisite for subsequent transfer of ENA to ischemic cells.

The pre-experiment demonstrated that FUS stimulating pEGFP-C1-ACPNs cavitation damaged the endothelial lining and efficaciously increased vessel permeability to EB (Supplementary Fig. 6A). Apparently, the EB extravasation was positively related to the ultrasonic energy and effective FUS irradiation time (Supplementary Fig. 6B), but a higher ultrasonic parameter of 3.9-5-50-1 caused bleeding due to vascular damage (Supplementary Fig. 6A). Additionally, EB extravasation did not indicate the exosmosis of ACPNs that showed a larger size requiring a greater degree of BBB opening. Therefore, to efficiently achieve controlled delivery of ENA to ischemic cells in vivo, the ACPN penetration and diffusion in brain parenchyma was firstly explored through the opened BBB obtained by FUS at 5 parameters referred to in vitro experimental parameters.

**Fig. 6:**
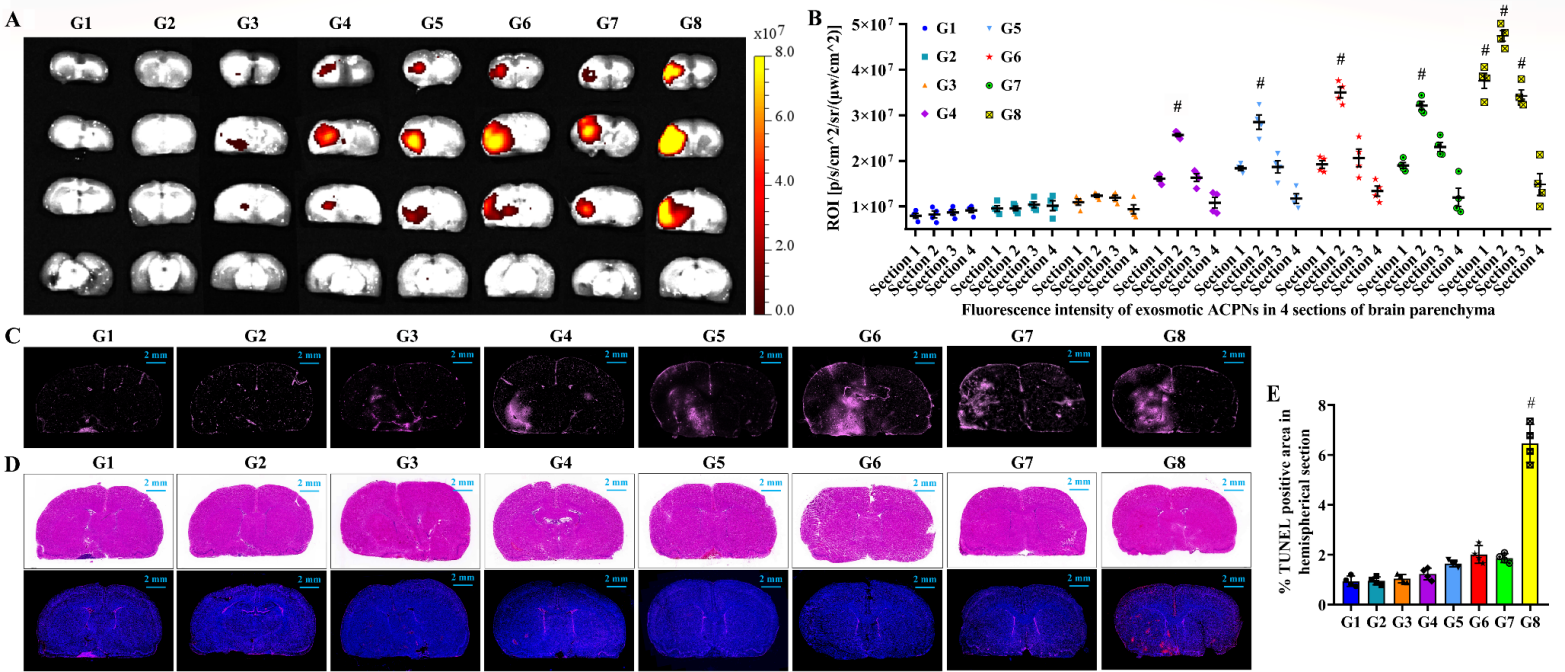
The analysis of RB-ACPN extravasation into the ischemic brain parenchyma through the opened BBB following the first ultrasonic cavitation. **A-B** ILLIS imaging to observe the RB fluorescently labeled ACPNs in brain, and their fluorescence intensity analysis of RB-ACPNs in left hemisphere. **C** fluorescence images of RB-ACPNs in brain sections detected by slide scanning system. **D** HE and TUNEL staining. **E** analysis of TUNEL positive area ratio. G1, tMCAO; G2, tMCAO + pre-ACPNs; G3, tMCAO + ACPNs; G4, tMCAO + ACPNs + FUS (3.3-20-200-5); G5, tMCAO + ACPNs + FUS (3.3-60-200-9); G6, tMCAO + ACPNs + FUS (3.6-20-100-7); G7, tMCAO + ACPNs + FUS (3.6-40-200-3); G8, tMCAO + ACPNs + FUS (3.9-5-50-1). The ultrasonic parameters are depicted as PNP-PRF-PD-ET, of which the unit is MPa-Hz-cycles-s. Note: RB-ACPNs, RB-tagged pEGFP-C1-ACPNs. #, indicating that the data are significantly higher than the other treatment groups (*p* < 0.05), and significantly higher than the control group (*p* < 0.001). Four biological replicates were designed for each experimental group

The fluorescence images of RB-labeled ACPNs in brain sections detected by ILLIS and slide scanning system revealed that FUS stimulating RB-ACPNs cavitation opened BBB to promote the extravasation of the nonactivated RB-ACPNs to the ischemic brain parenchyma (Fig. 6A-C). It also confirmed that the cavitation dose or FUS intensity was directly correlated to the level of BBB opening for RB-ACPN penetration, which was consistent with previously reported results [64, 65]. Moreover, RB fluorescence in the ischemic lesions of the tMCAO + ACPN group was increased as compared with that in the tMCAO group and tMCAO + pre-ACPN group (Fig. 6A-C), suggesting that pVEC dramatically enhanced the accumulation of RB-ACPNs in the brain parenchyma across the BBB. However, the advantage of pVEC in driving ACPNs across the BBB was not fully developed, as reported in the study of Stalmans S et al. [66], which may be attributed to the large weight or diameter of ACPNs.

It is controversial the fact that no extravasation of nonactivated RB-ACPNs to the ischemic brain parenchyma was observed, but infiltration of free RB-cholesterol detached from RB-ACPNs that was driven to phase transition and cavitation under FUS exposure, as shown in Fig. 6A-C. This hypothesis was verified by treating tMCAO rats with an intravenous injection of 0.25 mL ACPNs and irradiating them with FUS at 3.3-20-200-5 parameters to open BBB, followed by an immediate injection of 0.25 mL RB-ACPNs and 2 h buffer period to allow RB-ACPN extravasation. ILLIS images (Supplementary Fig. 7A) and fluorescence intensity analysis (Supplementary Fig. 7B) of RB-ACPNs in brain coronal sections proved that RB-ACPNs effectively permeated through the opened BBB to ischemic brain parenchyma, which was considered as direct evidence of RB-ACPNs rather than RB-cholesterol extravasation through the opened BBB.

Generally, the ACPN phase-shifted microbubbles oscillated and even collapsed under FUS exposure, which was supposed to induce sufficient forces on the endothelium through either radiation and shear forces induced by microstreaming around the microbubbles (Fig. 1B; Supplementary Fig. 5) [67–70]. Additionally, Chen CC et al. reported [54] that, although the utilization of nanodroplets to mediate FUS-induced BBB opening achieved a homogeneous dextran delivery within the targeted hippocampus, they presented a higher BBB opening pressure threshold than microbubbles, which might cause unrepairable mechanical damage. However, HE and TUNEL staining did not show significant tissue variation and cell apoptosis after FUS stimulating ACPN cavitation to open BBB at parameters of 3.3-20-200-5, 3.3-60-200-9, 3.6-20-100-7 and 3.6-40-200-3 (Fig. 6C-E). Additionally, the loss of ACPNs due to phase transition-cavitation increased with greater FUS intensity, thus our recommendation is to open BBB in vivo applying the parameters of 3.3-20-200-5 (Supplementary Fig. 5), which also avoided vascular injury to the maximum extent.

#### Second ultrasonic cavitation controlled IS-targeted ENA delivery

Sonoporation was suitable for site-specific gene delivery, since the permeabilization of the vasculature and controlled release or delivery of the cargo only occurred at the sites where ultrasound was applied and where there were artificial cavitation nuclei [62]. After ACPNs penetrated through the cerebrovascular wall into the ischemic brain parenchyma, the cell membrane created the next physical barrier repressing ENA entry. Then, the second FUS stimulated pericellular ACPN phase-shifted to microbubbles, and the latter oscillated and even collapsed, the mechanical force of which induced sonoporation thus enhancing cell permeability to ENA (Fig. 1C; Supplementary Fig. 5) [48, 71, 72]. As regards the FUS parameters applied for ENA controlled delivery in vitro 3D hydrogel and BBB opening in vivo, the feasibility of ultrasonic cavitation facilitating ENA liberation and delivery in vivo was further verified. In view of ENA delivery in 3D hydrogel did not involve the sacrifice of some large-sized ACPNs during BBB opening; the ultrasonic parameters 3.3-60-200-9 or 3.6-20-100-7 were applied for FAM-eNA or pEGFP-C1 controlled delivery, respectively, in tMCAO rats after using 3.3-20-200-5 to open BBB.

Usually, normal brain tissue, tMCAO brain tissue, and tMCAO + FUS brain tissue were used as the controls. However, in the preliminary experiment, no evident variance in fluorescence intensity was observed between the left and right hemispheres of three types of brain tissue (Supplementary Fig. 8). Although pVEC promoted a few ACPN extravasation into the brain parenchyma (Fig. 6A-C; Supplementary Fig. 7), nonactivated ACPNs did not deliver ENA to cells without FUS exposure (Fig. 5D, F). Therefore, taking into account the experimental prerequisites and animal welfare guidelines, only one group (tMCAO + ACPNs + FUS) was considered in the follow-up experiments, and the ENA delivery efficiency in the left ischemic brain parenchyma was directly compared with that in the right hemisphere. It is noteworthy that precision diagnosis and treatment is the focus of our medical research, especially because the brain implemented with therapeutic measure must be under strict monitoring (including transcranial Doppler, computed tomography angiography, and magnetic resonance angiography) to ensure that only specific lesions are performed. However, in this work, the left hemisphere of tMCAO rat model was almost completely infarcted (Supplementary Fig. 8A), which indicated that it was only necessary to move the FUS focal region to locate and cover the designated region of the left hemisphere under B-mode ultrasound guidance and visual inspection.

**Fig. 7:**
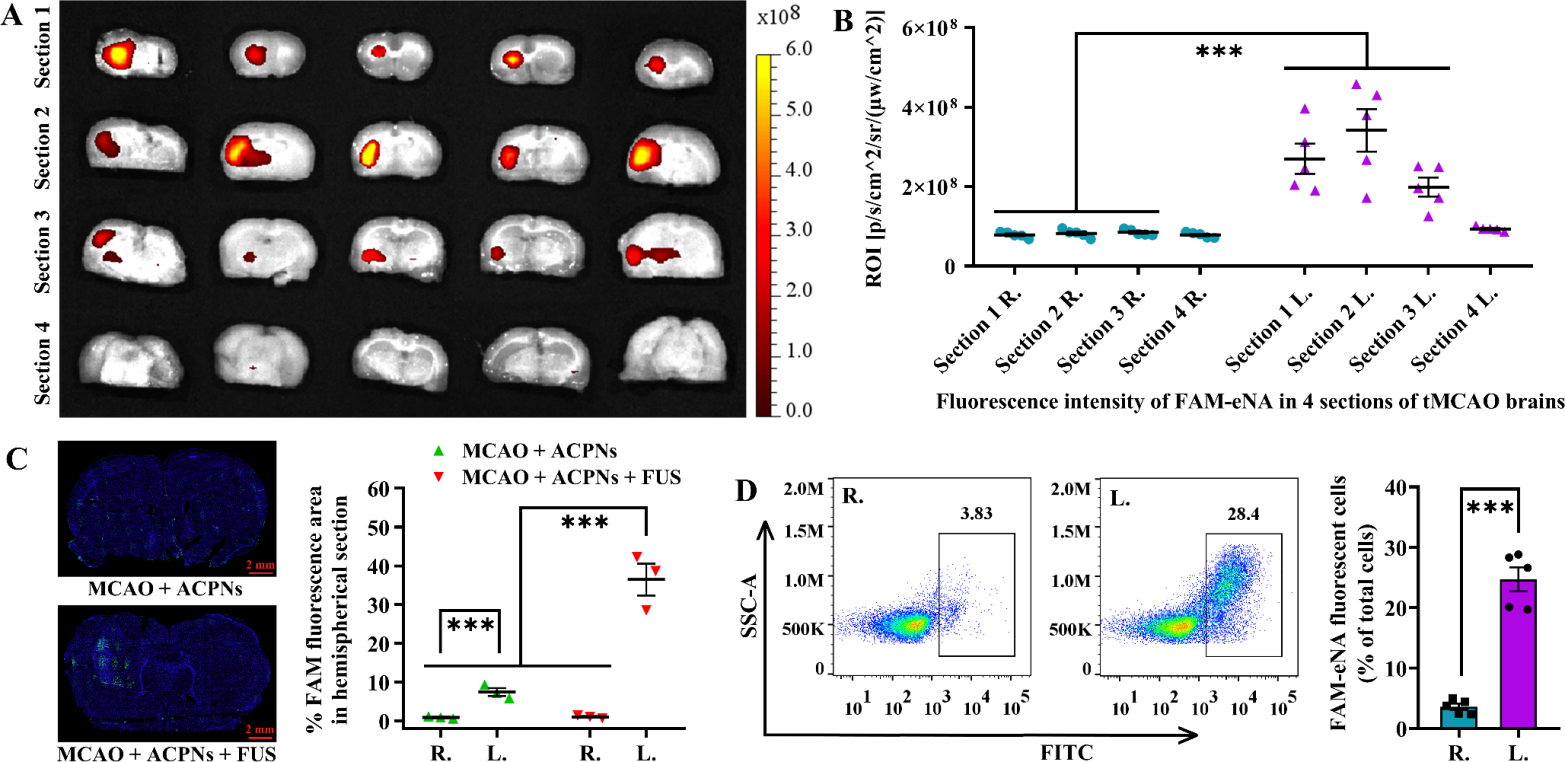
Second FUS stimulating ACPNs cavitation for FAM-eNA controlled delivery to ischemic cells in tMCAO models. **A-B** FAM-eNA fluorescence imaging through ILLIS in 4 coronal slices of brain tissues, and fluorescence intensity analysis corresponding to the images. **C** slide scanning images of FAM fluorescent cells in coronal slices, and statistical analysis of the FAM fluorescent area ratio. **D** flow cytometry analysis of FAM labeled fluorescent cells. R., the right side of brain section; L., the left side of brain slice. Each experimental group was treated with 5 biological replicates

In terms of FAM-eNA controlled delivery, ILLIS and slide scanning images demonstrated that the FAM fluorescence intensity in the left hemisphere was significantly higher than that in the right hemisphere (Fig. 7A-C), albeit it was a circumstantial evidence due to the fact that FAM-eNA displayed both intracellular and extracellular fluorescence. Most strikingly, the flow cytometry results directly proved that the number of FAM fluorescent cells was significantly higher in the parenchyma of the left ischemic hemisphere (24.62 ± 2.57%) as compared to the right hemisphere (3.61 ± 1.57%) (Fig. 7D). In summary, the above results fully confirmed the effective controlled delivery of FAM-eNA in tMCAO lesion by the second FUS stimulating ACPN cavitation at parameters of 3.3 MPa, 60 Hz, 200 cycles and 9 s.

**Fig. 8:**
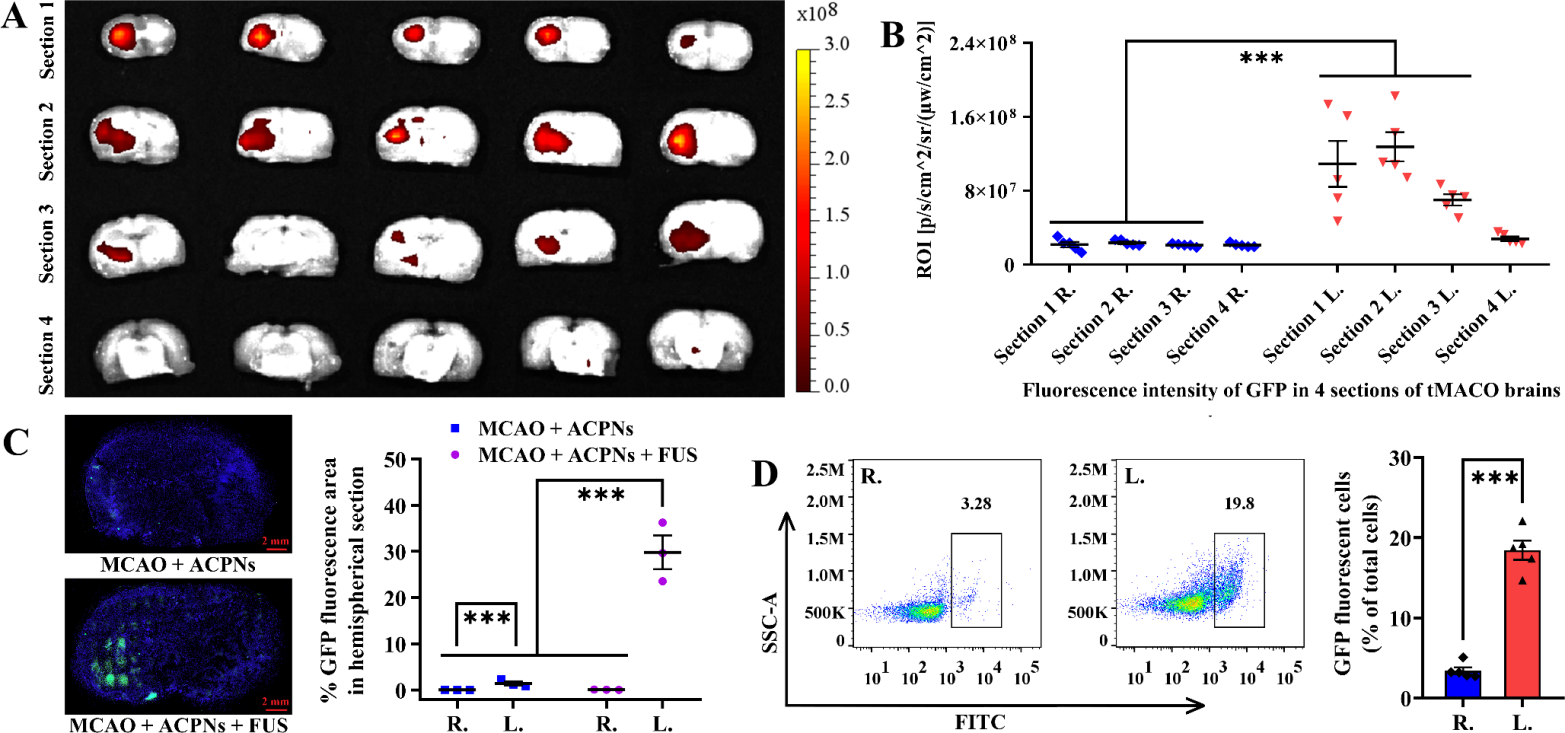
Second FUS stimulating ACPNs cavitation for pEGFP-C1 delivery to ischemic cells in tMCAO models. **A-B** GFP fluorescence images exported from the ILLIS, and the fluorescence intensity analysis corresponding to the ILLIS images. **C** slide scanning images of GFP fluorescent cells in brain coronal slices, and statistical analysis of the positive GFP fluorescent area ratio. **D** flow cytometry analysis of GFP fluorescent cells. R., the right side of brain section; L., the left side of brain slice. Each experimental group was treated with 5 biological replicates

There is an essential difference between pEGFP-C1 and FAM-eNA, that was only evident when pEGFP-C1 entered cells expressing GFP protein exhibiting fluorescence under excitation light. ILLIS imaging and tissue section scanning of GFP fluorescence proved that FUS plus ACPNs efficiently controlled pEGFP-C1 delivery to the ischemic cells in the parenchyma of the left hemisphere in comparison to the right hemisphere (Fig. 8A-C). Particularly, the flow cytometry results more precisely confirmed that 18.63 ± 2.13% GFP fluorescent cells in the parenchyma of the left ischemic hemisphere were significantly higher than 2.86 ± 1.58% in the right hemisphere (Fig. 8D). Additionally, HE and TUNEL staining showed no significant cell damage between the left and right cerebral hemispheres after two-step ultrasonic cavitation controlling FAM-eNA or pEGFP-C1 delivery at parameters of 3.3-60-200-9 or 3.6-20-200-7 respectively (Supplementary Fig. 9).

ACPN phase-shifted microbubbles showed repetitive contraction and expansion upon 3.3-60-200-9 lower intensity of FUS irradiation that created microstreaming in the nearby medium, which produced shear forces to the cell permeability for the delivery of short fragments of FAM-eNA (Supplementary Fig. 5) [73, 74]. When FUS intensity exceeded a certain level, many microbubbles oscillated more vigorously and collapsed strenuously, generating forceful shock waves and microjets, of which powerful stresses created pores within the cell membrane to enhance the paracellular permeability of pEGFP-C1 (Supplementary Fig. 5) [75]. It should be noted that the specific ultrasonic parameters proposed above for the second FUS stimulating ACPN cavitation to deliver FAM-eNA or pEGFP-C1 are important guidelines but not absolute indicators. For example, paragraph 3.2.3 showed that the application of 3.6-40-200-3 and 3.6-60-200-4 ultrasonic parameters delivered pEGFP-C1 more efficiently than the 3.6-20-100-7. Although the ultrasonic intensity was 3.6 MPa, the duty cycle or actual sonication time of 3.6-40-200-3 and 3.6-60-200-4 was higher than that of 3.6-20-100-7. Under the precondition of higher pEGFP-C1 delivery efficiency, 3.6-20-100-7 should be selected to minimize the potential damage to neuroglial cells as much as possible, while higher parameters should be selected for the delivery of ENA with larger molecular weight according to the above study.

## Conclusions

The objective of this research was to investigate the feasibility of two-step ultrasonic cavitation controlling micromolecular or macromolecular ENA delivery in ischemic brain parenchyma of tMCAO rats for IS gene therapy. FUS plus ACPNs, as a novel strategy for ENA controlled delivery, firstly relied on the feasibility of FUS stimulating ACPN phase-shifted microbubbles to noninvasively, transiently and reversibly open BBB to facilitate the extravasation of nonactivated ACPNs to the interstitial space of the ischemic brain parenchyma, whose procedure was mainly attributed to ultrasonic parameters or cavitation dose. Our conclusion after comparison and screening was that first FUS stimulated ACPN cavitation at the parameters of 3.3 MPa, 20 Hz, 200 cycles and 5 s, which was optimal for BBB opening to enhance nonactivated ACPN extravasation.

Then, it was followed by a second FUS stimulating ACPN cavitation in the ischemic brain parenchyma for controlling the ENA delivery to cells, which was significantly affected by FUS parameters. As verified by ILLIS, LCFM and flow cytometry, the second ultrasonic cavitation effectively controlled ENA delivery in ischemic brain parenchyma, particularly applying the ultrasonic parameters of 3.3 MPa, 60 Hz, 200 cycles and 9 s for controlling FAM-eNA delivery, and 3.6 MPa, 20 Hz, 200 cycles and 7 s for pEGFP-C1 controlled delivery, which minimized the unintended adverse bioeffects or even undesired brain damage.

## Electronic supplementary material

Below is the link to the electronic supplementary material.

Supplementary Material 1

Supplementary Material 2

Supplementary Material 3

Supplementary Material 4

Supplementary Material 5

Supplementary Material 6

Supplementary Material 7

Supplementary Material 8

Supplementary Material 9

Supplementary Material 10

Supplementary Material 11

## Data Availability

The authors confirm that the data supporting the findings of this study are available within the article and its supplementary materials.

## References

[1] Liu Y, Yin B, Cong Y. The probability of ischaemic stroke prediction with a Multi-Neural-Network model. Sens (Basel). 2020;20(17). 10.3390/s20174995. PubMed PMID: 32899242; PubMed Central PMCID: PMCPMC7506623. Epub 2020/09/10. 10.3390/s20174995 PMC7506623 32899242

[2] Peng JW, Nfor ON, Ho CC, Hsu SY, Lung CC, Tantoh DM, et al. Interactive association between CYP2C9 rs2860905 polymorphism and atrial fibrillation on ischemic stroke in Taiwan biobank participants. Pharmgenomics Pers Med. 2021;14:1087–92. 10.2147/pgpm.S310675. Epub 2021/09/14. 34511979 10.2147/PGPM.S310675 PMC8418368

[3] Fan PL, Wang SS, Chu SF, Chen NH. Time-dependent dual effect of microglia in ischemic stroke. Neurochem Int. 2023;169:105584. 10.1016/j.neuint.2023.105584. Epub 2023/07/17. 37454817 10.1016/j.neuint.2023.105584

[4] Li XH, Yin FT, Zhou XH, Zhang AH, Sun H, Yan GL, et al. The signaling pathways and targets of natural compounds from traditional Chinese medicine in treating ischemic stroke. Molecules. 2022;27(10). 10.3390/molecules27103099. PubMed PMID: 35630576; PubMed Central PMCID: PMCPMC9148018. Epub 2022/05/29. 10.3390/molecules27103099 PMC9148018 35630576

[5] Saini V, Guada L, Yavagal DR. Global epidemiology of stroke and access to acute ischemic stroke interventions. Neurology. 2021;97(20 Suppl 2):S6–16. 10.1212/wnl.0000000000012781. Epub 2021/11/18. 34785599 10.1212/WNL.0000000000012781

[6] Rikhtegar R, Yousefi M. Stem cell-based cell therapy for neuroprotection in stroke: A review. 2019;120(6):8849–62. 10.1002/jcb.28207. PubMed PMID: 30506720. 10.1002/jcb.28207 30506720

[7] Jolugbo P, Ariëns RAS. Thrombus composition and efficacy of thrombolysis and thrombectomy in acute ischemic stroke. Stroke. 2021;52(3):1131–42. 10.1161/strokeaha.120.032810. Epub 2021/02/11. 33563020 10.1161/STROKEAHA.120.032810 PMC7610448

[8] Feske SK. Ischemic stroke. Am J Med. 2021;134(12):1457–64. 10.1016/j.amjmed.2021.07.027. 34454905 10.1016/j.amjmed.2021.07.027

[9] Lapergue B, Blanc R, Costalat V, Desal H, Saleme S, Spelle L, Effect of Thrombectomy With Combined Contact Aspiration and Stent Retriever vs Stent Retriever Alone on Revascularization in Patients With Acute Ischemic Stroke and Large Vessel Occlusion. The ASTER2 Randomized Clinical Trial. Jama. 2021;326(12):1158-69. Epub 2021/09/29. 10.1001/jama.2021.13827. PubMed PMID: 34581737; PubMed Central PMCID: PMCPMC8479584 Neurovascular, Penumbra, Balt, and MicroVention during the conduct of the study; and nonfinancial support for travel from Medtronic and personal fees for speaker honoraria from Penumbra outside the submitted work. Dr Costalat reported grants from Medronic, Stryker, Cerenovus, Balt, and MicroVention outside the submitted work. Dr Spelle reported personal fees from MicroVention, Medtronic, and Balt; and other (hospital grant) from Philips outside the submitted work. Dr Marnat reported personal fees (for paid lectures) from Medtronic and MicroVention outside the submitted work. Dr Eugene reported personal fees from Biomodex outside the submitted work. Dr Mazighi reported personal fees (for consulting) from Acticor Biotech, Air Liquide, Boerhinger Ingelheim, Servier, Medtronic, and Amgen; and personal fees (for paid lectures) from Servier, Amgen, Medtronic, and AstraZeneca outside the submitted work. Dr Bracard reports personal fees from General Electric Medical Systems and nonfinancial support from MicroVention Europe outside the submitted work. Dr Renaud reported grants from the French health ministry Programme Hospitalier Recherche Clinique (PHRC), MicroVention, Penumbra, and Stryker during the conduct of the study. Dr Piotin received institutional grants from Stryker, Medtronic, MicroVention and Balt outside the submitted work. No other disclosures were reported.

[10] Szentirmai O, Carter BS. Genetic and cellular therapies for cerebral infarction. Neurosurgery. 2004;55(2):283–6. discussion 96– 7. 10.1227/01.neu.0000129681.85731.00. Epub 2004/07/24. 15271234 10.1227/01.neu.0000129681.85731.00

[11] Tiedt S, Dichgans M. Role of Non-Coding RNAs in Stroke. Stroke. 2018;49(12):3098– 106. Epub 2018/12/21. 10.1161/strokeaha.118.021010. PubMed PMID: 30571439. 10.1161/STROKEAHA.118.021010 30571439

[12] Heydari E, Alishahi M, Ghaedrahmati F, Winlow W, Khoshnam SE. The role of non-coding RNAs in neuroprotection and angiogenesis following ischemic stroke. 2020;35(1):31–43. 10.1007/s11011-019-00485-2. PubMed PMID: 31446548. 10.1007/s11011-019-00485-2 31446548

[13] Jeyaseelan K, Lim KY, Armugam A. MicroRNA expression in the blood and brain of rats subjected to transient focal ischemia by middle cerebral artery occlusion. Stroke. 2008;39(3):959–66. 10.1161/STROKEAHA.107.500736. PubMed PMID: 18258830. 18258830 10.1161/STROKEAHA.107.500736

[14] Liu FJ, Lim KY, Kaur P, Sepramaniam S, Armugam A, Wong PT, et al. MicroRNAs involved in regulating spontaneous recovery in embolic stroke model. PLoS ONE. 2013;8(6):e66393. 10.1371/journal.pone.0066393. PubMed PMID: 23823624; PubMed Central PMCID: PMC3688919. 23823624 10.1371/journal.pone.0066393 PMC3688919

[15] Li P, Teng F, Gao F, Zhang M, Wu J, Zhang C. Identification of Circulating MicroRNAs as potential biomarkers for detecting acute ischemic stroke. Cell Mol Neurobiol. 2015;35(3):433–47. 10.1007/s10571-014-0139-5. PubMed PMID: 25410304. 25410304 10.1007/s10571-014-0139-5 PMC11486203

[16] Jickling GC, Ander BP, Zhan X, Noblett D, Stamova B, Liu D. MicroRNA expression in peripheral blood cells following acute ischemic stroke and their predicted gene targets. PLoS ONE. 2014;9(6):e99283. 10.1371/journal.pone.0099283. PubMed PMID: 24911610; PubMed Central PMCID: PMC4050059. 24911610 10.1371/journal.pone.0099283 PMC4050059

[17] Sorensen SS, Nygaard AB, Nielsen MY, Jensen K, Christensen T. MiRNA expression profiles in cerebrospinal fluid and blood of patients with acute ischemic stroke. Transl Stroke Res. 2014;5(6):711–8. 10.1007/s12975-014-0364-8. PubMed PMID: 25127724. 25127724 10.1007/s12975-014-0364-8

[18] Bernardo BC, Ooi JY, Lin RC, McMullen JR. MiRNA therapeutics: a new class of drugs with potential therapeutic applications in the heart. Future Med Chem. 2015;7(13):1771–92. 10.4155/fmc.15.107. PubMed PMID: 26399457. 26399457 10.4155/fmc.15.107

[19] Ebert MS, Sharp PA. MicroRNA sponges: progress and possibilities. RNA. 2010;16(11):2043–50. 10.1261/rna.2414110. PubMed PMID: 20855538; PubMed Central PMCID: PMC2957044. 20855538 10.1261/rna.2414110 PMC2957044

[20] Chuapoco MR, Flytzanis NC, Goeden N, Christopher Octeau J, Roxas KM, Chan KY, et al. Adeno-associated viral vectors for functional intravenous gene transfer throughout the non-human primate brain. Nat Nanotechnol. 2023;18(10):1241–51. 10.1038/s41565-023-01419-x. Epub 2023/07/11. 37430038 10.1038/s41565-023-01419-x PMC10575780

[21] Shay TF, Sullivan EE, Ding X, Chen X, Ravindra Kumar S, Goertsen D, et al. Primate-conserved carbonic anhydrase IV and murine-restricted LY6C1 enable blood-brain barrier crossing by engineered viral vectors. Sci Adv. 2023;9(16):eadg6618. 10.1126/sciadv.adg6618. Epub 2023/04/19. 37075114 10.1126/sciadv.adg6618 PMC10115422

[22] Hacein-Bey-Abina S, Von Kalle C, Schmidt M, McCormack MP, Wulffraat N, Leboulch P, et al. LMO2-associated clonal T cell proliferation in two patients after gene therapy for SCID-X1. Science. 2003;302(5644):415–9. 10.1126/science.1088547. Epub 2003/10/18. 14564000 10.1126/science.1088547

[23] Raper SE, Chirmule N, Lee FS, Wivel NA, Bagg A, Gao GP et al. Fatal systemic inflammatory response syndrome in a ornithine transcarbamylase deficient patient following adenoviral gene transfer. Molecular genetics and metabolism. 2003;80(1–2):148– 58. Epub 2003/10/22. doi: 10.1016/j.ymgme.2003.08.016. PubMed PMID: 14567964. 10.1016/j.ymgme.2003.08.016 14567964

[24] Check E. A tragic setback. Nature. 2002;420(6912):116-8. Epub 2002/11/15. 10.1038/420116a. PubMed PMID: 12432357. 10.1038/420116a 12432357

[25] Ogris M, Brunner S, Schuller S, Kircheis R, Wagner E. PEGylated DNA/transferrin-PEI complexes: reduced interaction with blood components, extended circulation in blood and potential for systemic gene delivery. Gene Ther. 1999;6(4):595–605. 10.1038/sj.gt.3300900. Epub 1999/09/07. 10476219 10.1038/sj.gt.3300900

[26] Liu Y, Li Y, Keskin D. Poly(beta-Amino Esters): Synthesis, Formulations, and Their Biomedical Applications. 2019;8(2):e1801359. 10.1002/adhm.201801359. PubMed PMID: 30549448. 10.1002/adhm.201801359 30549448

[27] Itaka K, Ishii T, Hasegawa Y, Kataoka K. Biodegradable polyamino acid-based polycations as safe and effective gene carrier minimizing cumulative toxicity. Biomaterials. 2010;31(13):3707–14. PubMed PMID: 20153891. 20153891 10.1016/j.biomaterials.2009.11.072

[28] Lin S, Du F, Wang Y, Ji S, Liang D, Yu L, et al. An acid-labile block copolymer of PDMAEMA and PEG as potential carrier for intelligent gene delivery systems. Biomacromolecules. 2008;9(1):109–15. 10.1021/bm7008747. Epub 2007/12/20. 18088093 10.1021/bm7008747

[29] Xiu KM, Yang JJ, Zhao NN, Li JS, Xu FJ. Multiarm cationic star polymers by atom transfer radical polymerization from beta-cyclodextrin cores: influence of arm number and length on gene delivery. Acta Biomater. 2013;9(1):4726–33. 10.1016/j.actbio.2012.08.020. Epub 2012/08/25. 22917804 10.1016/j.actbio.2012.08.020

[30] Sato T, Ishii T, Okahata Y. In vitro gene delivery mediated by Chitosan. Effect of pH, serum, and molecular mass of Chitosan.on the transfection efficiency. Biomaterials. 2001;22(15):2075–80. 10.1016/s0142-9612(00)00385-9. Epub 2001/07/04. 11432586 10.1016/s0142-9612(00)00385-9

[31] Yang Z, Gao D, Cao Z, Zhang C, Cheng D, Liu J et al. Drug and gene co-delivery systems for cancer treatment. Biomaterials science. 2015;3(7):1035-49. Epub 2015/07/30. 10.1039/c4bm00369a. PubMed PMID: 26221938. 10.1039/c4bm00369a 26221938

[32] Poon C, McMahon D, Hynynen K. Noninvasive and targeted delivery of therapeutics to the brain using focused ultrasound. Neuropharmacology. 2017;120:20–37. 10.1016/j.neuropharm.2016.02.014. Epub 2016/02/26. 26907805 10.1016/j.neuropharm.2016.02.014 PMC5028296

[33] Aryal M, Arvanitis CD, Alexander PM, McDannold N. Ultrasound-mediated blood-brain barrier disruption for targeted drug delivery in the central nervous system. Adv Drug Deliv Rev. 2014;72:94–109. 10.1016/j.addr.2014.01.008. Epub 2014/01/28. 24462453 10.1016/j.addr.2014.01.008 PMC4041837

[34] Ferrara K, Pollard R, Borden M. Ultrasound microbubble contrast agents: fundamentals and application to gene and drug delivery. Annu Rev Biomed Eng. 2007;9:415–47. 10.1146/annurev.bioeng.8.061505.095852. Epub 2007/07/27. 17651012 10.1146/annurev.bioeng.8.061505.095852

[35] Stratmeyer ME, Greenleaf JF, Dalecki D, Salvesen KA. Fetal ultrasound: mechanical effects. Journal of ultrasound in medicine: official journal of the American Institute of ultrasound in medicine. 2008;27(4):597–605; quiz 6–9. Epub 2008/03/25. 10.7863/jum.2008.27.4.597. PubMed PMID: 18359910. 10.7863/jum.2008.27.4.597 18359910

[36] Schlicher RK, Radhakrishna H, Tolentino TP, Apkarian RP, Zarnitsyn V, Prausnitz MR. Mechanism of intracellular delivery by acoustic cavitation. Ultrasound Med Biol. 2006;32(6):915–24. 10.1016/j.ultrasmedbio.2006.02.1416. Epub 2006/06/21. 16785013 10.1016/j.ultrasmedbio.2006.02.1416

[37] Liu Y, Miyoshi H, Nakamura M. Encapsulated ultrasound microbubbles: therapeutic application in drug/gene delivery. J Control Release. 2006;114(1):89–99. 10.1016/j.jconrel.2006.05.018. Epub 2006/07/11. 16824637 10.1016/j.jconrel.2006.05.018

[38] Kodama T, Tomita Y, Koshiyama K, Blomley MJ. Transfection effect of microbubbles on cells in superposed ultrasound waves and behavior of cavitation bubble. Ultrasound Med Biol. 2006;32(6):905–14. 10.1016/j.ultrasmedbio.2006.03.004. Epub 2006/06/21. 16785012 10.1016/j.ultrasmedbio.2006.03.004

[39] Sheikov N, McDannold N, Vykhodtseva N, Jolesz F, Hynynen K. Cellular mechanisms of the blood-brain barrier opening induced by ultrasound in presence of microbubbles. Ultrasound Med Biol. 2004;30(7):979–89. 10.1016/j.ultrasmedbio.2004.04.010. Epub 2004/08/18. 15313330 10.1016/j.ultrasmedbio.2004.04.010

[40] Tung YS, Vlachos F, Feshitan JA, Borden MA, Konofagou EE. The mechanism of interaction between focused ultrasound and microbubbles in blood-brain barrier opening in mice. J Acoust Soc Am. 2011;130(5):3059–67. PubMed PMID: 22087933; PubMed Central PMCID: PMCPmc3248062. 22087933 10.1121/1.3646905 PMC3248062

[41] Fan CH, Ting CY, Lin HJ, Wang CH, Liu HL, Yen TC, et al. SPIO-conjugated, doxorubicin-loaded microbubbles for concurrent MRI and focused-ultrasound enhanced brain-tumor drug delivery. Biomaterials. 2013;34(14):3706–15. 10.1016/j.biomaterials.2013.01.099. Epub 2013/02/26. 23433776 10.1016/j.biomaterials.2013.01.099

[42] Jordão JF, Thévenot E, Markham-Coultes K, Scarcelli T, Weng YQ, Xhima K, et al. Amyloid-β plaque reduction, endogenous antibody delivery and glial activation by brain-targeted, transcranial focused ultrasound. Exp Neurol. 2013;248:16–29. PubMed PMID: 23707300; PubMed Central PMCID: PMCPmc4000699. 23707300 10.1016/j.expneurol.2013.05.008 PMC4000699

[43] Jordão JF, Ayala-Grosso CA, Markham K, Huang Y, Chopra R, McLaurin J, et al. Antibodies targeted to the brain with image-guided focused ultrasound reduces amyloid-beta plaque load in the TgCRND8 mouse model of Alzheimer’s disease. PLoS ONE. 2010;5(5):e10549. 10.1371/journal.pone.0010549. Epub 2010/05/21. 20485502 10.1371/journal.pone.0010549 PMC2868024

[44] Huang Q, Deng J, Wang F, Chen S, Liu Y, Wang Z, et al. Targeted gene delivery to the mouse brain by MRI-guided focused ultrasound-induced blood-brain barrier disruption. Exp Neurol. 2012;233(1):350–6. 10.1016/j.expneurol.2011.10.027. Epub 2011/11/15. 22079586 10.1016/j.expneurol.2011.10.027

[45] Fan CH, Chang EL, Ting CY, Lin YC, Liao EC, Huang CY, et al. Folate-conjugated gene-carrying microbubbles with focused ultrasound for concurrent blood-brain barrier opening and local gene delivery. Biomaterials. 2016;106:46–57. 10.1016/j.biomaterials.2016.08.017. Epub 2016/08/22. 27544926 10.1016/j.biomaterials.2016.08.017

[46] Burgess A, Ayala-Grosso CA, Ganguly M, Jordão JF, Aubert I, Hynynen K. Targeted delivery of neural stem cells to the brain using MRI-guided focused ultrasound to disrupt the blood-brain barrier. PLoS ONE. 2011;6(11):e27877. 10.1371/journal.pone.0027877. Epub 2011/11/25. 22114718 10.1371/journal.pone.0027877 PMC3218061

[47] Dong W, Wu P, Zhou D, Huang J, Qin M, Yang X, et al. Ultrasound-Mediated gene therapy of hepatocellular carcinoma using Pre-microRNA Plasmid-Loaded nanodroplets. Ultrasound Med Biol. 2020;46(1):90–107. PubMed PMID: 31668943. 31668943 10.1016/j.ultrasmedbio.2019.09.016

[48] Dong W, Wu P, Qin M, Guo S, Liu H, Yang X, et al. Multipotent MiRNA Sponge-Loaded magnetic nanodroplets with Ultrasound/Magnet-Assisted delivery for hepatocellular carcinoma therapy. Mol Pharm. 2020;17(8):2891–910. 10.1021/acs.molpharmaceut.0c00336. Epub 2020/07/18. 32678617 10.1021/acs.molpharmaceut.0c00336

[49] Dong W, Huang A, Huang J, Wu P, Guo S, Liu H et al. Plasmid-loadable magnetic/ultrasound-responsive nanodroplets with a SPIO-NP dispersed perfluoropentane core and lipid shell for tumor-targeted intracellular plasmid delivery. Biomater Sci. 2020;8(19):5329-45. Epub 2020/08/15. 10.1039/d0bm00699h. PubMed PMID: 32793943. 10.1039/d0bm00699h 32793943

[50] Kofoed RH, Aubert I. Focused ultrasound gene delivery for the treatment of neurological disorders. Trends Mol Med. 2024. Epub 2024/01/13. 10.1016/j.molmed.2023.12.006. PubMed PMID: 38216449. 10.1016/j.molmed.2023.12.006 38216449

[51] Zhang X, Hu J, Zhao G, Huang N, Tan Y, Pi L, et al. PEGylated PLGA-based phase shift nanodroplets combined with focused ultrasound for blood brain barrier opening in rats. Oncotarget. 2017;8(24):38927–36. 10.18632/oncotarget.17155. Epub 2017/05/06. 28473660 10.18632/oncotarget.17155 PMC5503583

[52] Sirsi S, Feshitan J, Kwan J, Homma S, Borden M. Effect of microbubble size on fundamental mode high frequency ultrasound imaging in mice. Ultrasound Med Biol. 2010;36(6):935– 48. Epub 2010/05/08. doi: 10.1016/j.ultrasmedbio.2010.03.015. PubMed PMID: 20447755; PubMed Central PMCID: PMCPmc2878876. 10.1016/j.ultrasmedbio.2010.03.015 PMC2878876 20447755

[53] Cavalli R, Bisazza A, Lembo D. Micro- and nanobubbles: a versatile non-viral platform for gene delivery. Int J Pharm. 2013;456(2):437–45. PubMed PMID: 24008081. 24008081 10.1016/j.ijpharm.2013.08.041

[54] Chen CC, Sheeran PS, Wu SY, Olumolade OO, Dayton PA, Konofagou EE. Targeted drug delivery with focused ultrasound-induced blood-brain barrier opening using acoustically-activated nanodroplets. J Control Release. 2013;172(3):795–804. PubMed PMID: 24096019; PubMed Central PMCID: PMCPMC3866692. 24096019 10.1016/j.jconrel.2013.09.025 PMC3866692

[55] Wu SY, Fix SM, Arena CB, Chen CC, Zheng W, Olumolade OO, et al. Focused ultrasound-facilitated brain drug delivery using optimized nanodroplets: vaporization efficiency dictates large molecular delivery. Phys Med Biol. 2018;63(3):035002. 10.1088/1361-6560/aaa30d. Epub 2017/12/21. 29260735 10.1088/1361-6560/aaa30d PMC5823501

[56] Lea-Banks H, Meng Y, Wu SK, Belhadjhamida R, Hamani C, Hynynen K. Ultrasound-sensitive nanodroplets achieve targeted neuromodulation. J Control Release. 2021;332:30–9. 10.1016/j.jconrel.2021.02.010. Epub 2021/02/19. 33600879 10.1016/j.jconrel.2021.02.010 PMC8089063

[57] Shen Y, Guo J, Chen G, Chin CT, Chen X, Chen J, et al. Delivery of liposomes with different sizes to mice brain after sonication by focused ultrasound in the presence of microbubbles. Ultrasound Med Biol. 2016;42(7):1499–511. 10.1016/j.ultrasmedbio.2016.01.019. Epub 2016/04/30. 27126236 10.1016/j.ultrasmedbio.2016.01.019

[58] Wang DS, Panje C, Pysz MA, Paulmurugan R, Rosenberg J, Gambhir SS, et al. Cationic versus neutral microbubbles for ultrasound-mediated gene delivery in cancer. Radiology. 2012;264(3):721–32. 10.1148/radiol.12112368. Epub 2012/06/23. 22723497 10.1148/radiol.12112368 PMC3426857

[59] Panje CM, Wang DS, Pysz MA, Paulmurugan R, Ren Y, Tranquart F, et al. Ultrasound-mediated gene delivery with cationic versus neutral microbubbles: effect of DNA and microbubble dose on in vivo transfection efficiency. Theranostics. 2012;2(11):1078–91. 10.7150/thno.4240. Epub 2012/12/12. 23227124 10.7150/thno.4240 PMC3516840

[60] Shpak O, Verweij M, Vos HJ, de Jong N, Lohse D, Versluis M. Acoustic droplet vaporization is initiated by superharmonic focusing. Proc Natl Acad Sci U S A. 2014;111(5):1697–702. 10.1073/pnas.1312171111. Epub 2014/01/23. 24449879 10.1073/pnas.1312171111 PMC3918756

[61] Lin CY, Pitt WG. Acoustic droplet vaporization in biology and medicine. Biomed Res Int. 2013;2013:404361. Epub 2013/12/19. doi: 10.1155/2013/404361. PubMed PMID: 24350267; PubMed Central PMCID: PMCPmc3853706. 24350267 10.1155/2013/404361 PMC3853706

[62] Sirsi SR, Hernandez SL, Zielinski L, Blomback H, Koubaa A, Synder M, et al. Polyplex-microbubble hybrids for ultrasound-guided plasmid DNA delivery to solid tumors. J Control Release. 2012;157(2):224–34. 10.1016/j.jconrel.2011.09.071. PubMed PMID: 21945680; PubMed Central PMCID: PMC3822338. 21945680 10.1016/j.jconrel.2011.09.071 PMC3822338

[63] Toyoda K, Chu Y, Heistad DD. Gene therapy for cerebral vascular disease: update 2003. Br J Pharmacol. 2003;139(1):1–9. 10.1038/sj.bjp.0705217. Epub 2003/05/15. 12746217 10.1038/sj.bjp.0705217 PMC1573819

[64] Arvanitis CD, Livingstone MS, Vykhodtseva N, McDannold N. Controlled ultrasound-induced blood-brain barrier disruption using passive acoustic emissions monitoring. PLoS ONE. 2012;7(9):e45783. 10.1371/journal.pone.0045783. Epub 2012/10/03. 23029240 10.1371/journal.pone.0045783 PMC3454363

[65] Sheeran PS, Luois S, Dayton PA, Matsunaga TO. Formulation and acoustic studies of a new phase-shift agent for diagnostic and therapeutic ultrasound. Langmuir. 2011;27(17):10412–20. 10.1021/la2013705. Epub 2011/07/13. 21744860 10.1021/la2013705 PMC3164903

[66] Stalmans S, Bracke N, Wynendaele E, Gevaert B, Peremans K, Burvenich C, et al. Cell-Penetrating peptides selectively cross the Blood-Brain barrier in vivo. PLoS ONE. 2015;10(10):e0139652. 10.1371/journal.pone.0139652. Epub 2015/10/16. 26465925 10.1371/journal.pone.0139652 PMC4605843

[67] McDannold N, Vykhodtseva N, Hynynen K. Targeted disruption of the blood-brain barrier with focused ultrasound: association with cavitation activity. Phys Med Biol. 2006;51(4):793–807. 10.1088/0031-9155/51/4/003. Epub 2006/02/10. 16467579 10.1088/0031-9155/51/4/003

[68] Kinoshita M, McDannold N, Jolesz FA, Hynynen K. Targeted delivery of antibodies through the blood-brain barrier by MRI-guided focused ultrasound. Biochem Biophys Res Commun. 2006;340(4):1085–90. 10.1016/j.bbrc.2005.12.112. Epub 2006/01/13. 16403441 10.1016/j.bbrc.2005.12.112

[69] McDannold N, Vykhodtseva N, Raymond S, Jolesz FA, Hynynen K. MRI-guided targeted blood-brain barrier disruption with focused ultrasound: histological findings in rabbits. Ultrasound Med Biol. 2005;31(11):1527–37. 10.1016/j.ultrasmedbio.2005.07.010. Epub 2005/11/16. 16286030 10.1016/j.ultrasmedbio.2005.07.010

[70] Hynynen K, McDannold N, Sheikov NA, Jolesz FA, Vykhodtseva N. Local and reversible blood-brain barrier disruption by noninvasive focused ultrasound at frequencies suitable for trans-skull sonications. NeuroImage. 2005;24(1):12–20. 10.1016/j.neuroimage.2004.06.046. Epub 2004/12/14. 15588592 10.1016/j.neuroimage.2004.06.046

[71] Deckers R, Moonen CTW. Ultrasound triggered, image guided, local drug delivery. J Control Release. 2010;148(1):25–33. PubMed PMID: 20709123. 20709123 10.1016/j.jconrel.2010.07.117

[72] Ohl SW, Klaseboer E, Szeri AJ, Khoo BC. Lithotripter shock wave interaction with a bubble near various biomaterials. Phys Med Biol. 2016;61(19):7031–53. 10.1088/0031-9155/61/19/7031. Epub 2016/09/21. 27649337 10.1088/0031-9155/61/19/7031

[73] Liu HL, Fan CH, Ting CY, Yeh CK. Combining microbubbles and ultrasound for drug delivery to brain tumors: current progress and overview. Theranostics. 2014;4(4):432–44. 10.7150/thno.8074. Epub 2014/03/01. 24578726 10.7150/thno.8074 PMC3936295

[74] Sboros V. Response of contrast agents to ultrasound. Adv Drug Deliv Rev. 2008;60(10):1117–36. 10.1016/j.addr.2008.03.011. Epub 2008/05/20. PubMed PMID: 18486270. 18486270 10.1016/j.addr.2008.03.011

[75] Husseini GA, de la Diaz MA, Richardson ES, Christensen DA, Pitt WG. The role of cavitation in acoustically activated drug delivery. J Control Release. 2005;107(2):253–61. 10.1016/j.jconrel.2005.06.015. Epub 2005/07/28. 16046023 10.1016/j.jconrel.2005.06.015 PMC1409755

