# Supplementary material for: Two-step ultrasonic cavitation controlled delivery of brain exogenous nucleic acids for ischemic stroke using acoustic-cationic-polymeric-nanodroplets
Source: Drug Deliv Transl Res. 2025 Mar 6;15(10):3695–715. doi: 10.1007/s13346-025-01828-6 (PMC12397173; doi:10.1007/s13346-025-01828-6)
Supplement: Supplementary file 2 — Supplementary Material 2 [file 13346_2025_1828_MOESM2_ESM.docx]

**T****wo-step ultrasonic cavitation controlled delivery of brain exogenous nucleic acids for ischemic stroke using acoustic-cationic-polymeric-nanodroplets**


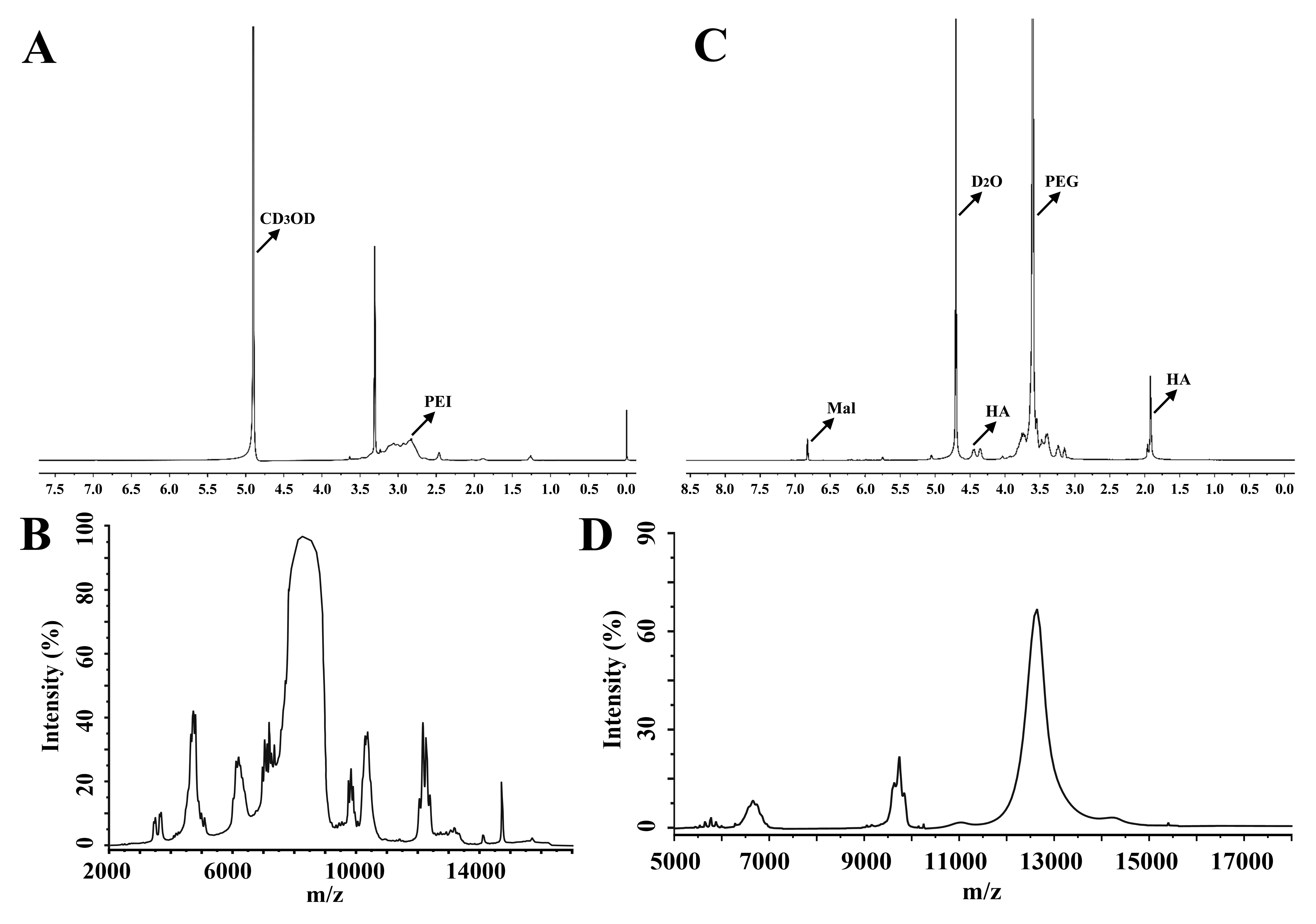


Supplementary Figure 1 1H NMR spectrum of C11F19-PEI and HA-PEG-MAL. A, 1H NMR spectrum of C11F19-PEI dissolved in methanol. B, MALDI-TOF MS of C11F19-PEI. C, 1H NMR spectrum of HA-PEG-MAL dissolved in water. D, MALDITOF MS of HA-PEG-MAL.


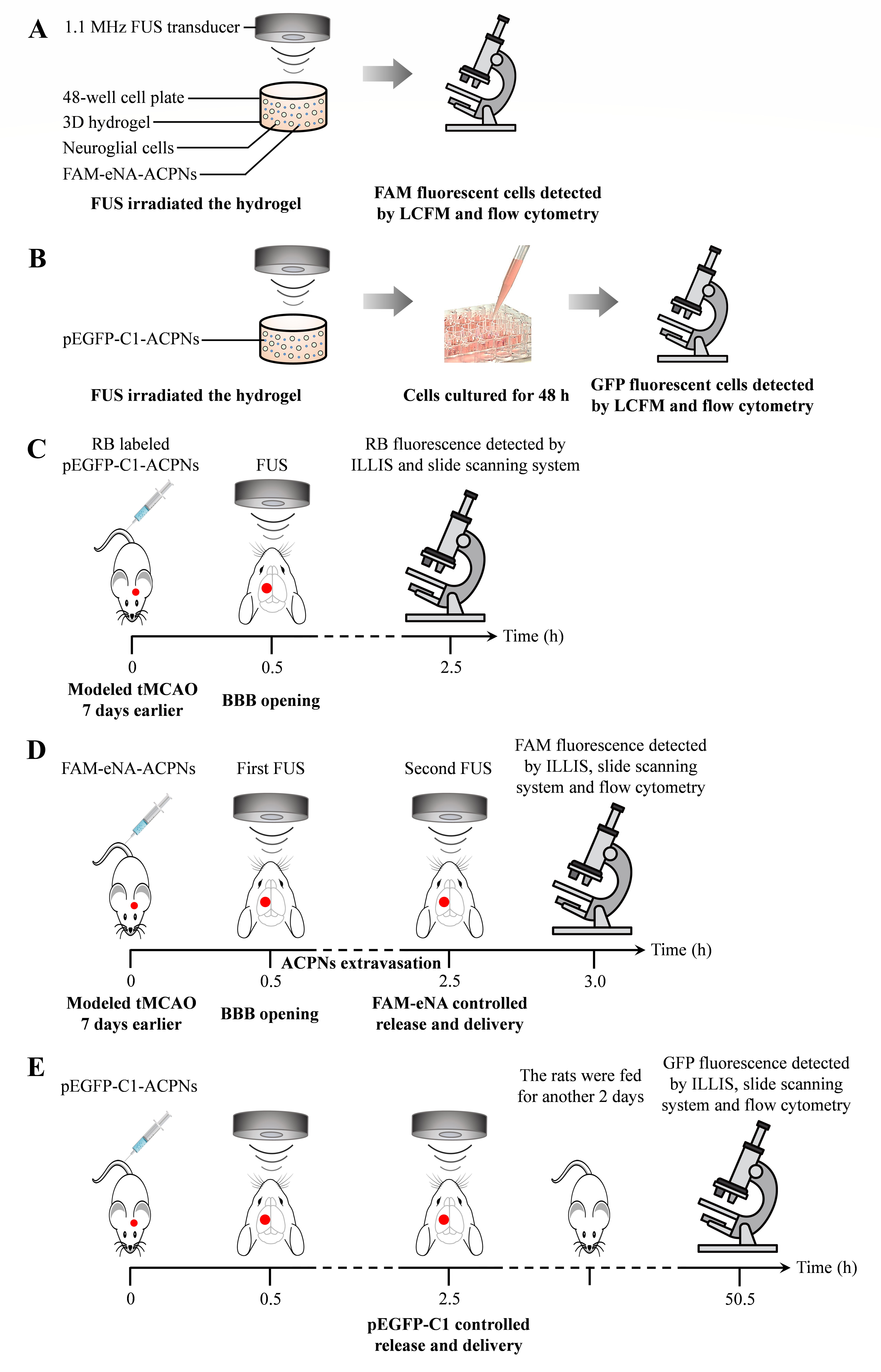


Supplementary Figure 2 Ultrasonic cavitation for controlling ENA delivery *in vitro* and *in vivo*. A, experimental process of FUS stimulating FAM-eNA-ACPNs cavitation for FAM-eNA delivery in 3D hydrogel. B, experimental procedure of FUS stimulating pEGFP-C1-ACPNs cavitation for pEGFP-C1 delivery in 3D hydrogel. C, experimental flow graph of FUS stimulating ACPNs cavitation for opening BBB to assist nonactivated ACPNs extravasation to ischemic brain parenchyma in tMCAO rats. D, experimental process of FUS stimulating FAM-eNA-ACPNs cavitation for controlling FAM-eNA delivery in tMCAO rats. E, experimental procedure of FUS stimulating pEGFP-C1-ACPNs cavitation for controlling pEGFP-C1 delivery in tMCAO rats.


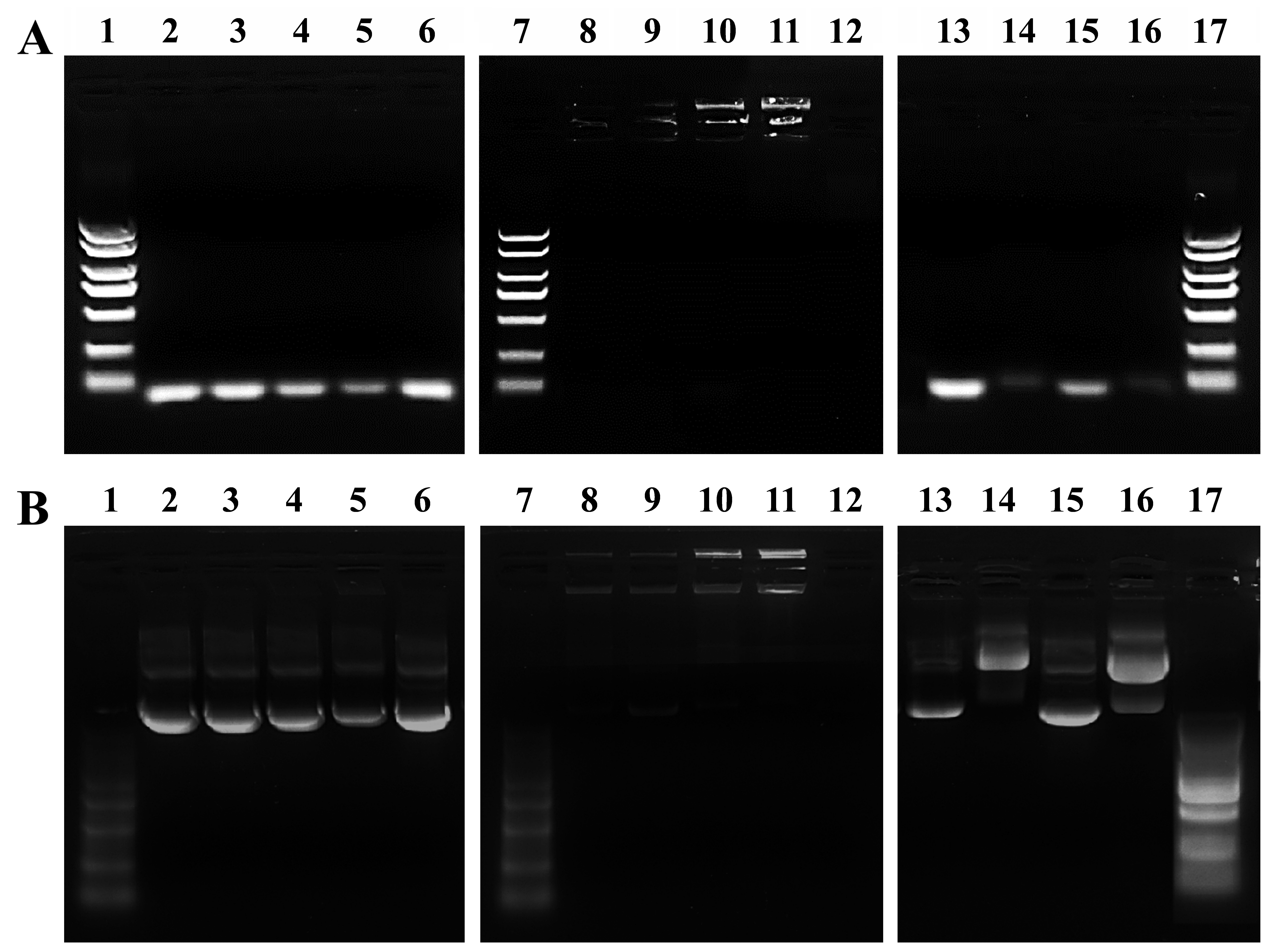


Supplementary Figure 3 Agarose gel electrophoresis to verify the feasibility of ACPNs carrying and protecting ENA. A, agarose gel electrophoresis image of the supernatant and precipitate of the mixture of FAM-eNA and I-ACPNs after centrifugation, and verify the ability of ACPNs in protecting FAM-eNA from degradation by nucleases in 10% serum. B, agarose gel electrophoresis image of the supernatant and precipitate of the compound of pEGFP-C1 and I-ACPNs after centrifugation, and confirm the protective effect of ACPNs to defend pEGFP-C1 against enzymolysis in 10% serum. 1, 7 and 17, the marker; 2-6, the supernatant of 5, 10, 20, 30, and 0 µL I-ACPNs mixed with 20 µL FAM-eNA (129.6 ng/µL) or pEGFP-C1 (159.87 ng/µL) respectively; 8-12, the sediment of 5, 10, 20, 30 and 0 µL I-ACPNs mixed with 20 µL FAM-eNA or pEGFP-C1 respectively; 13, the plasmids; 14, the plasmids in 10% serum; 15, the ACPNs in 10% serum; 16, the pri-ACPNs in 10% serum.


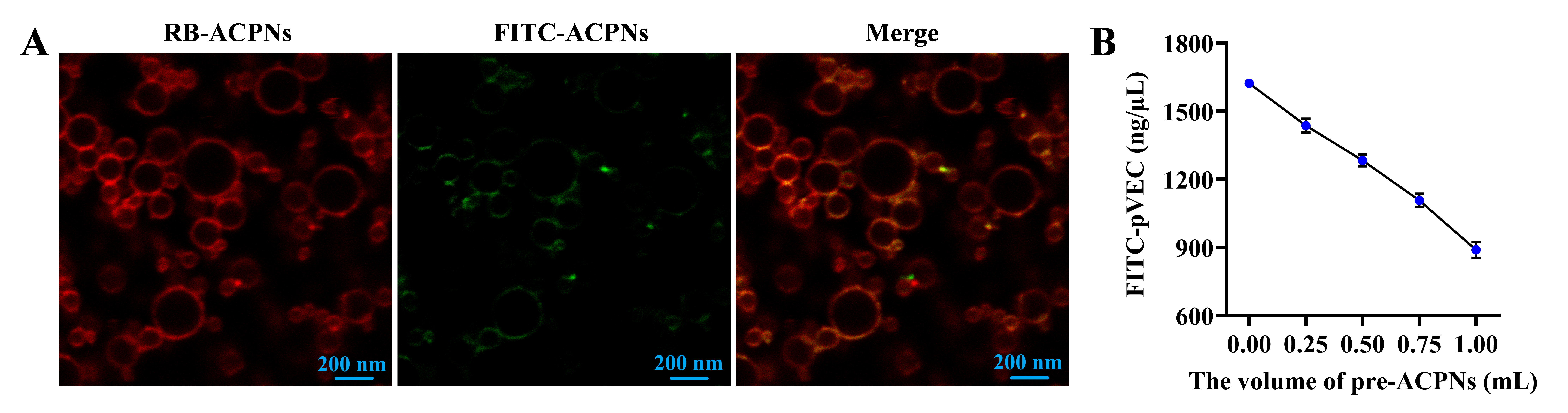


Supplementary Figure 4 Morphological characterization and FITC-pVEC coupled level of RB labled pEGFP-C1-ACPNs. A, LCFM images of RB labled pEGFP-C1-ACPNs. B, the coupled levels of FITC-pVEC on RB labled pEGFP-C1-ACPNs. Three replicates were designed for each experimental group.


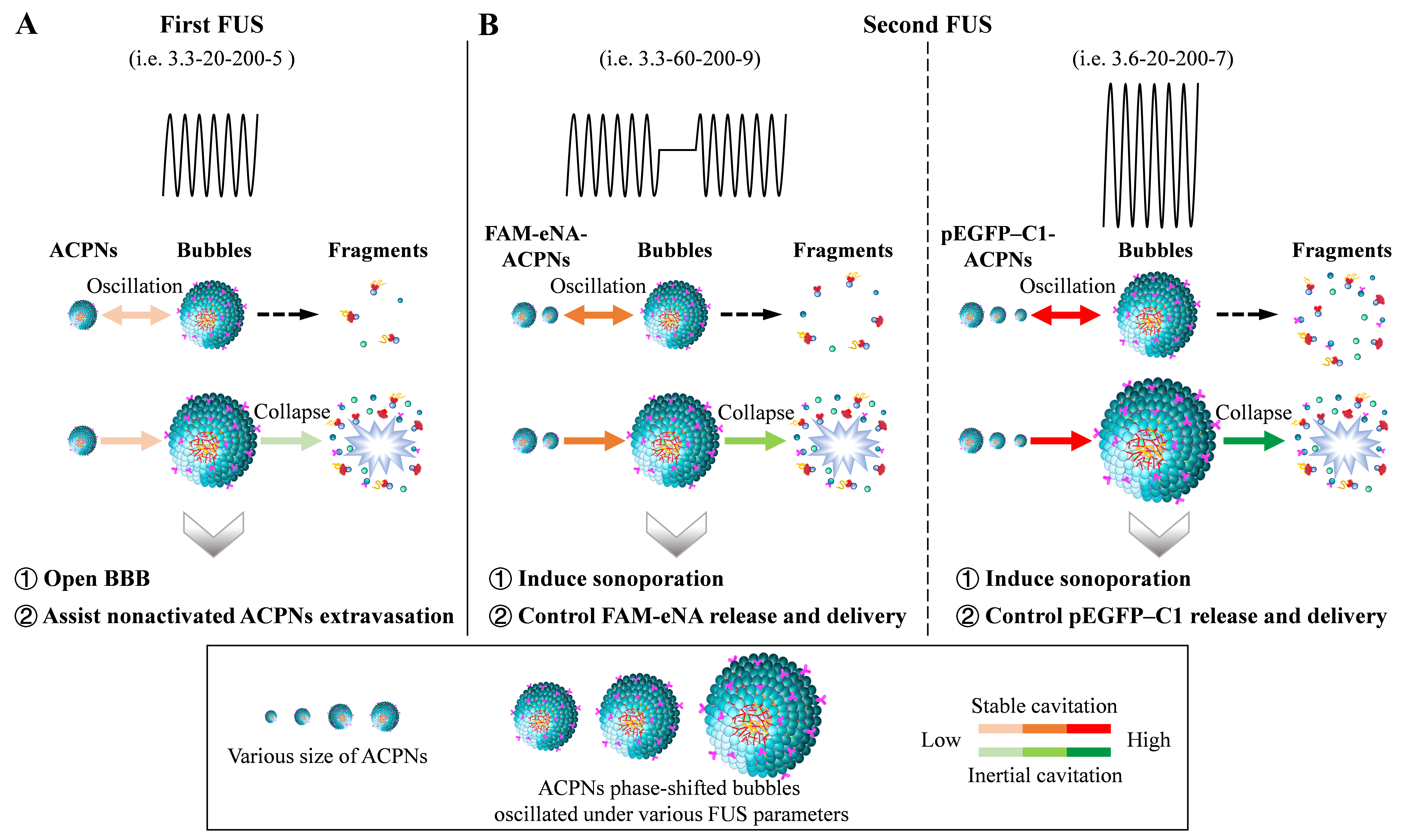


Supplementary Figure 5 Physical process analysis diagram of transcranial FUS stimulating ACPNs cavitation to open BBB, control ENA release and delivery. A, larger size ACPNs phase shifted to bubbles that undergo oscillation or collapse for BBB opening under low intensity of first FUS irradiation, which assisted nonactivated ACPNs extravasation. B, second FUS, at a higher intensity or longer sonication time, stimulated smaller ACPNs cavitation to induce sonoporation, control ENA release and delivery to ischemic cells.





Supplementary Figure 6 EB extravasation through the opened BBB via transcranial FUS stimulating pEGFP-C1-ACPNs cavitation. A, the images of EB extravasation in brain after FUS irradiation at different ultrasonic parameters. B, statistical analysis of the optical density to quantify EB extravasation. The ultrasonic parameters in the figure are labeled as PNP-PRF-PD-ET, of which the unit is MPa-Hz-cycles-s. Three biological replicates were designed for each experimental group.


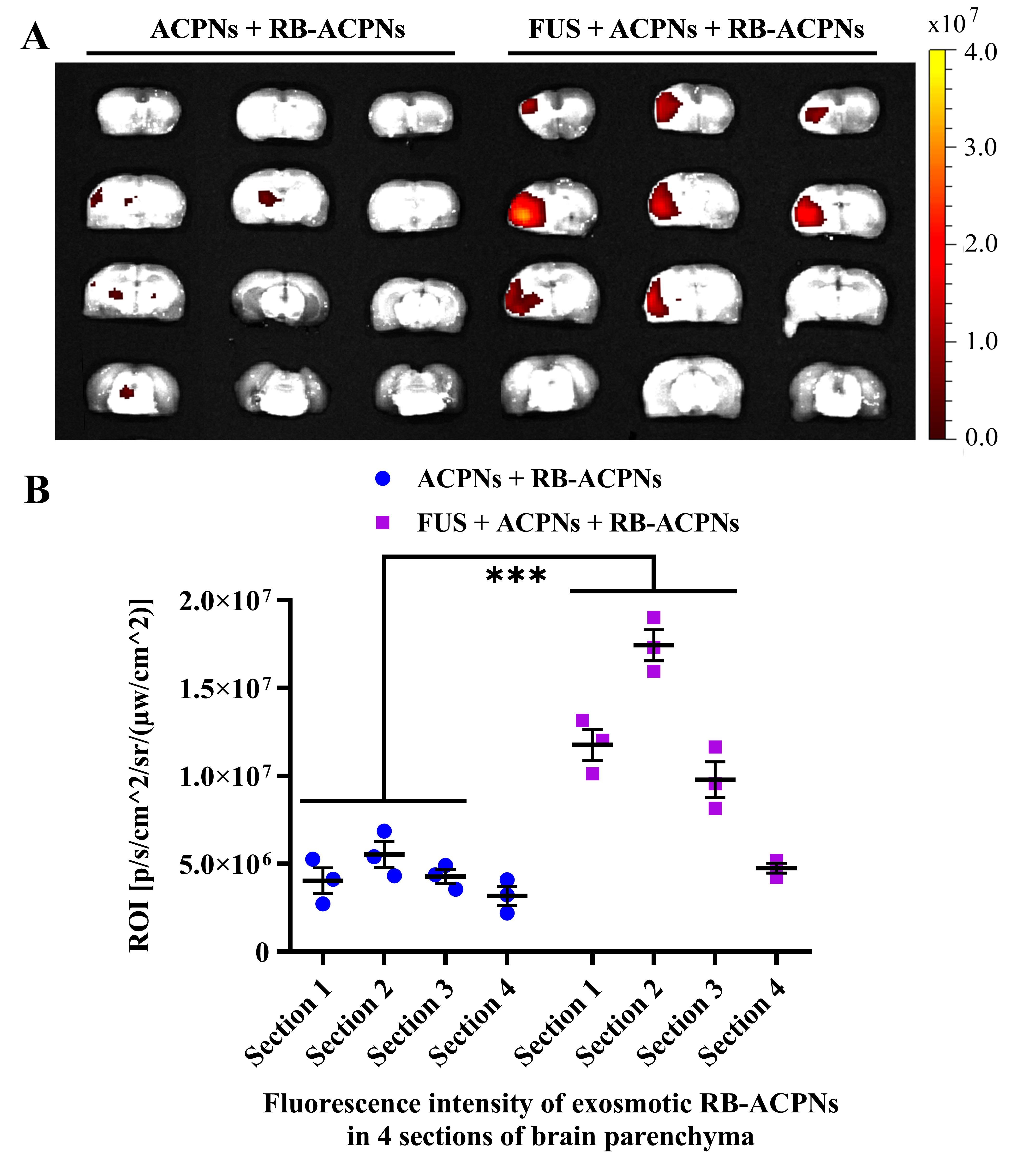


Supplementary Figure 7 RB-ACPNs permeation into ischemic brain parenchyma through the opened BBB following first ultrasonic cavitation. A, ILLIS images of RB-ACPNs extravasation into the ischemic lesion. B, fluorescence intensity analysis of RB-ACPNs in left hemisphere. Every experimental group received 3 biological duplicates of the treatment.


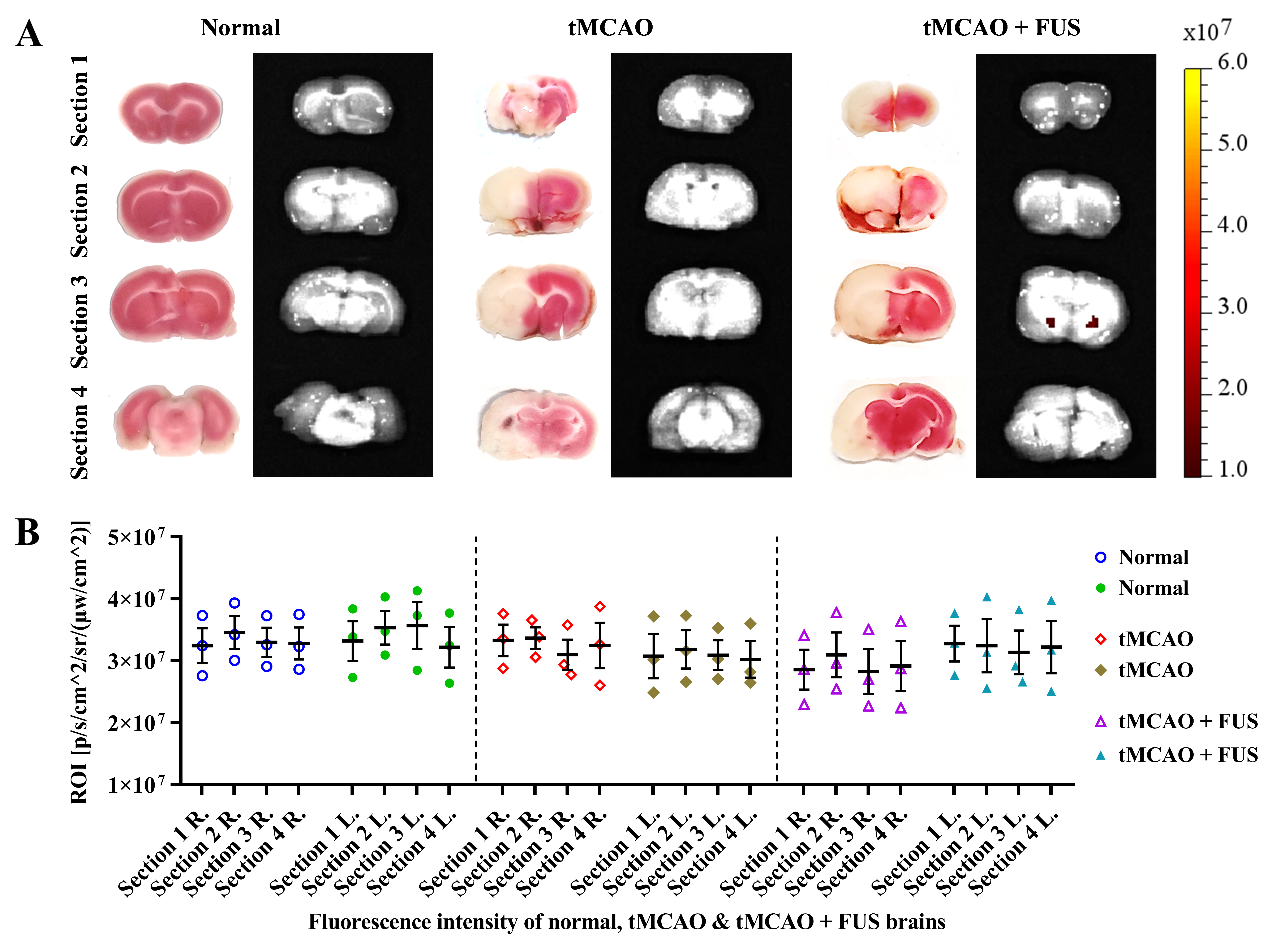


Supplementary Figure 8 ILLIS images and fluorescence intensity analysis of normal brain tissue, tMCAO brain tissue and tMCAO + FUS brain tissue. A, 4 coronal sections of brain tissue with equal longitudinal spacing performed with ILLIS imaging, and subjected to TTC staining. B, fluorescence intensity analysis of the ILLIS images. R., the right side of brain section; L., the left side of brain slice. Every experimental group received 3 biological duplicates of the treatment.


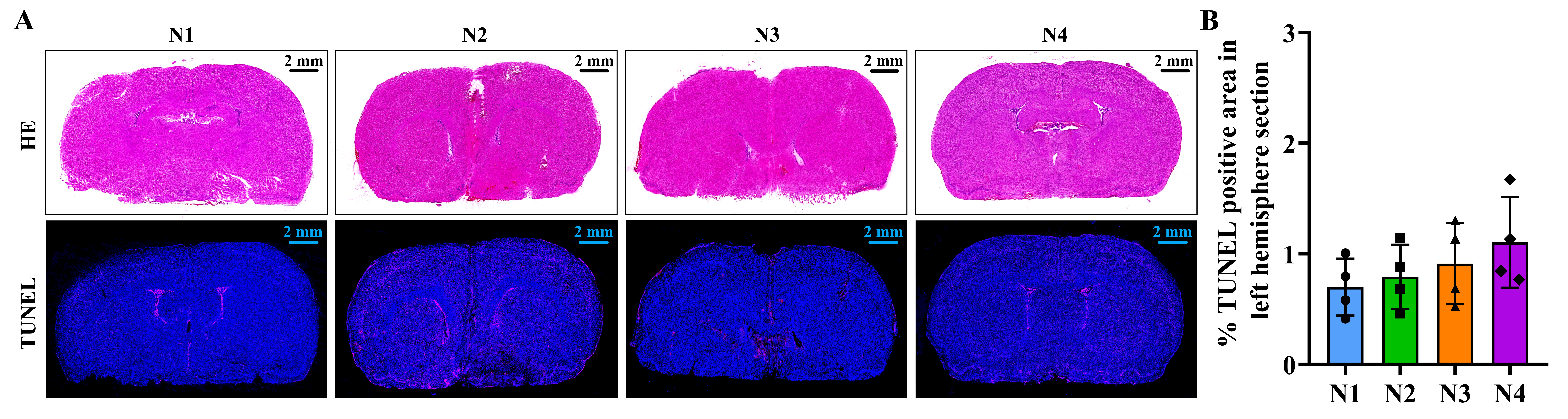


Supplementary Figure 9 HE and TUNEL staining of the brain tissue after two-step ultrasonic cavitation controlling FAM-eNA or pEGFP-C1 delivery at parameters of 3.3-60-200-9 or 3.6-20-200-7 respectively in tMCAO rats. A, images of HE and TUNEL staining. B, analysis of TUNEL positive area ratio in left hemisphere section. N1, tMCAO; N2, tMCAO + ACPNs; N3, tMCAO + FAM-eNA-ACPNs + FUS (3.3-60-200-9); N4, tMCAO + pEGFP-C1-ACPNs + FUS (3.6-20-200-7). Each experimental group involved 4 replicates.
