# Supplementary figures and images for: Two-step ultrasonic cavitation controlled delivery of brain exogenous nucleic acids for ischemic stroke using acoustic-cationic-polymeric-nanodroplets
Source: Drug Deliv Transl Res. 2025 Mar 6;15(10):3695–715. doi: 10.1007/s13346-025-01828-6 (PMC12397173; doi:10.1007/s13346-025-01828-6)

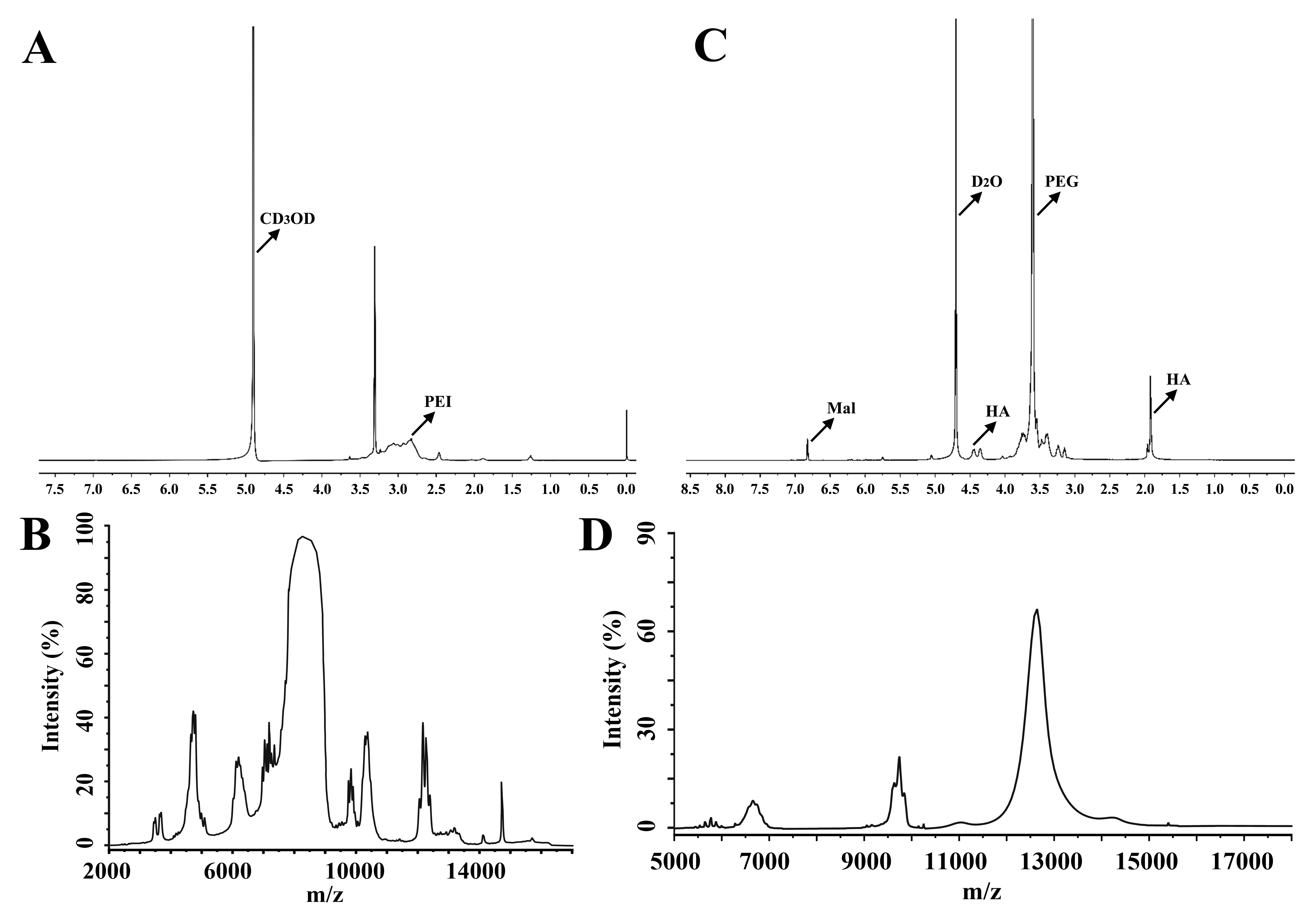

Supplement: Supplementary file 3 — Supplementary Material 3 [file 13346_2025_1828_MOESM3_ESM.tiff]

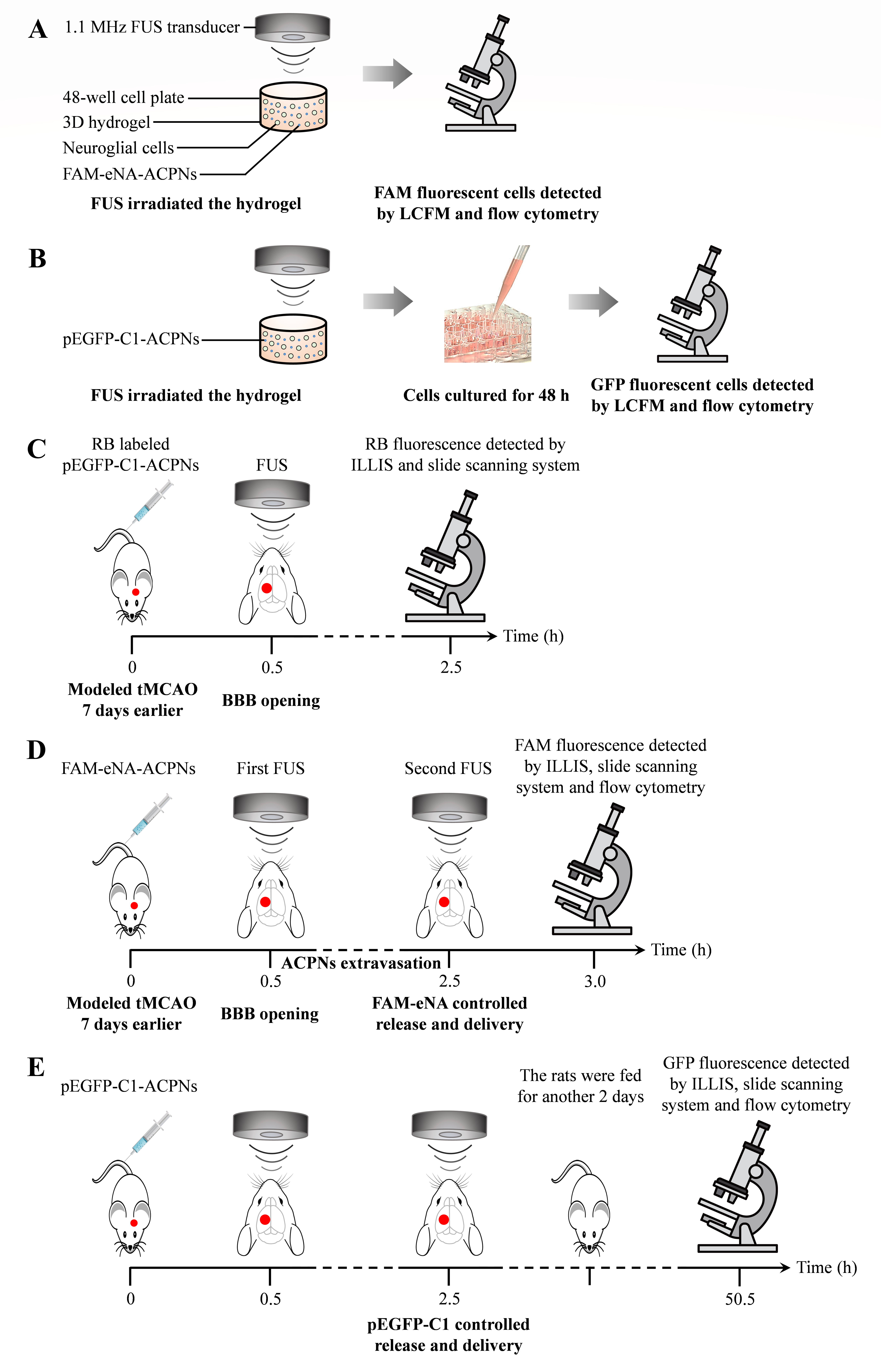

Supplement: Supplementary file 4 — Supplementary Material 4 [file 13346_2025_1828_MOESM4_ESM.tiff]

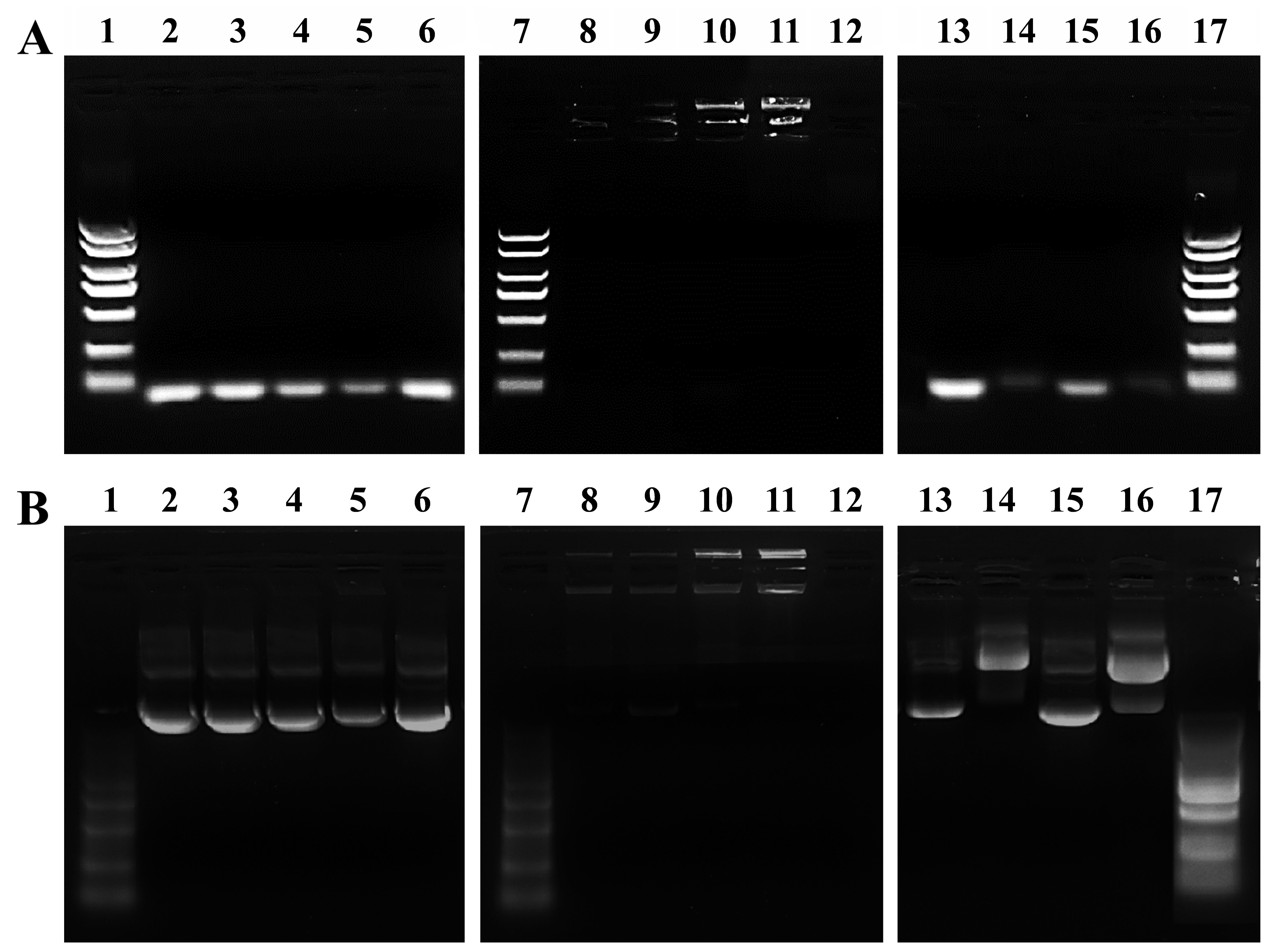

Supplement: Supplementary file 5 — Supplementary Material 5 [file 13346_2025_1828_MOESM5_ESM.tiff]

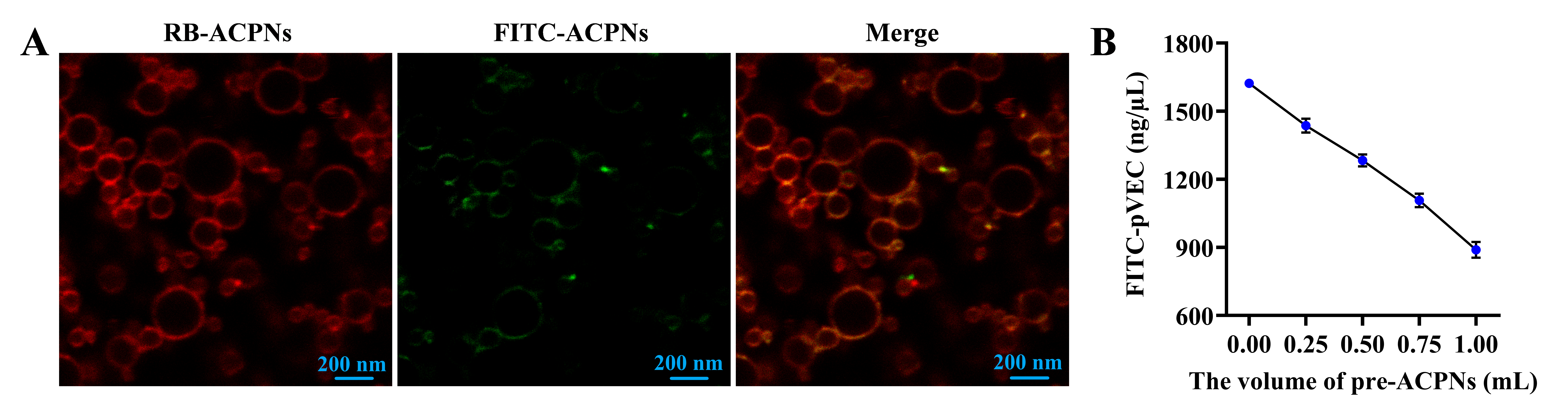

Supplement: Supplementary file 6 — Supplementary Material 6 [file 13346_2025_1828_MOESM6_ESM.tiff]

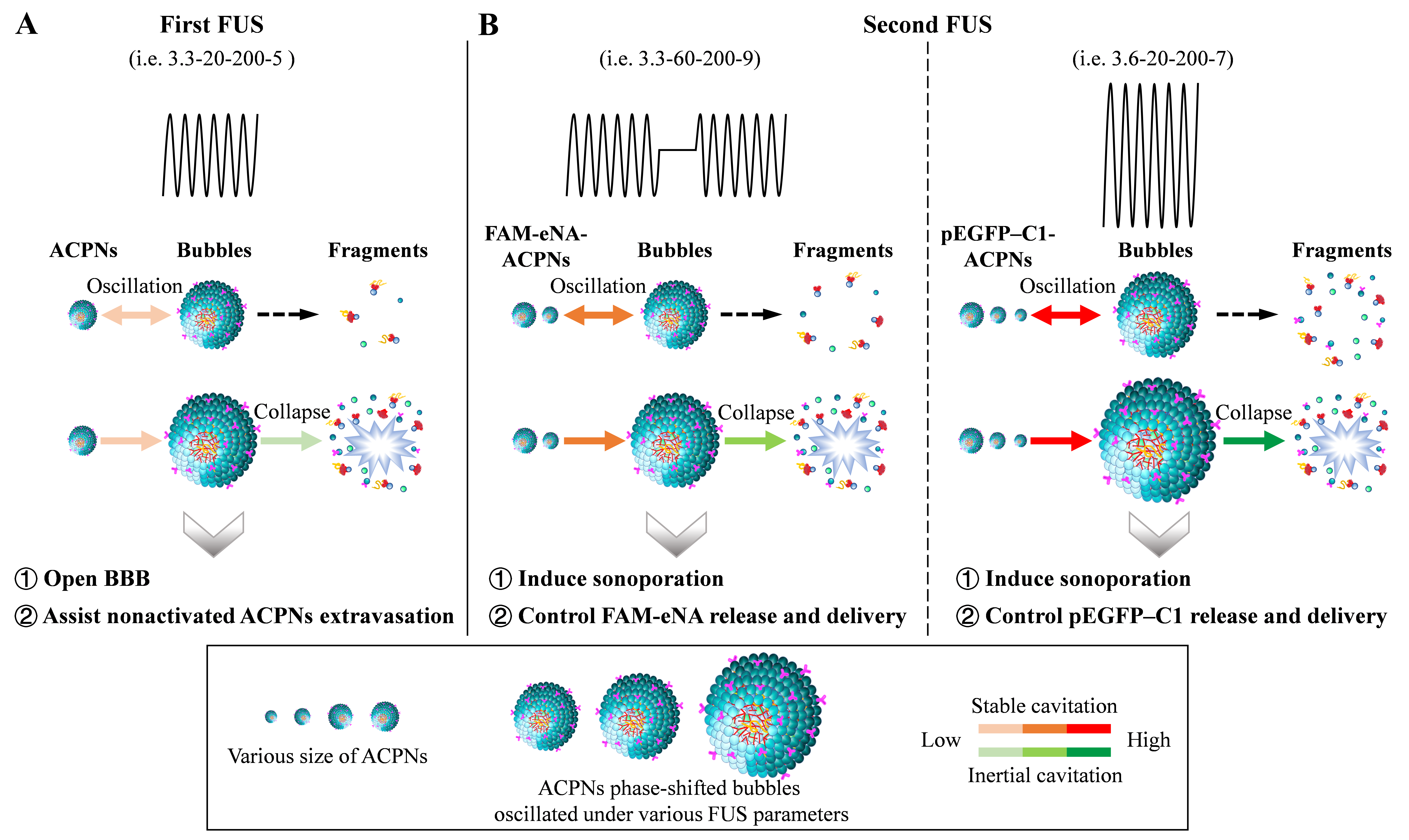

Supplement: Supplementary file 7 — Supplementary Material 7 [file 13346_2025_1828_MOESM7_ESM.tiff]

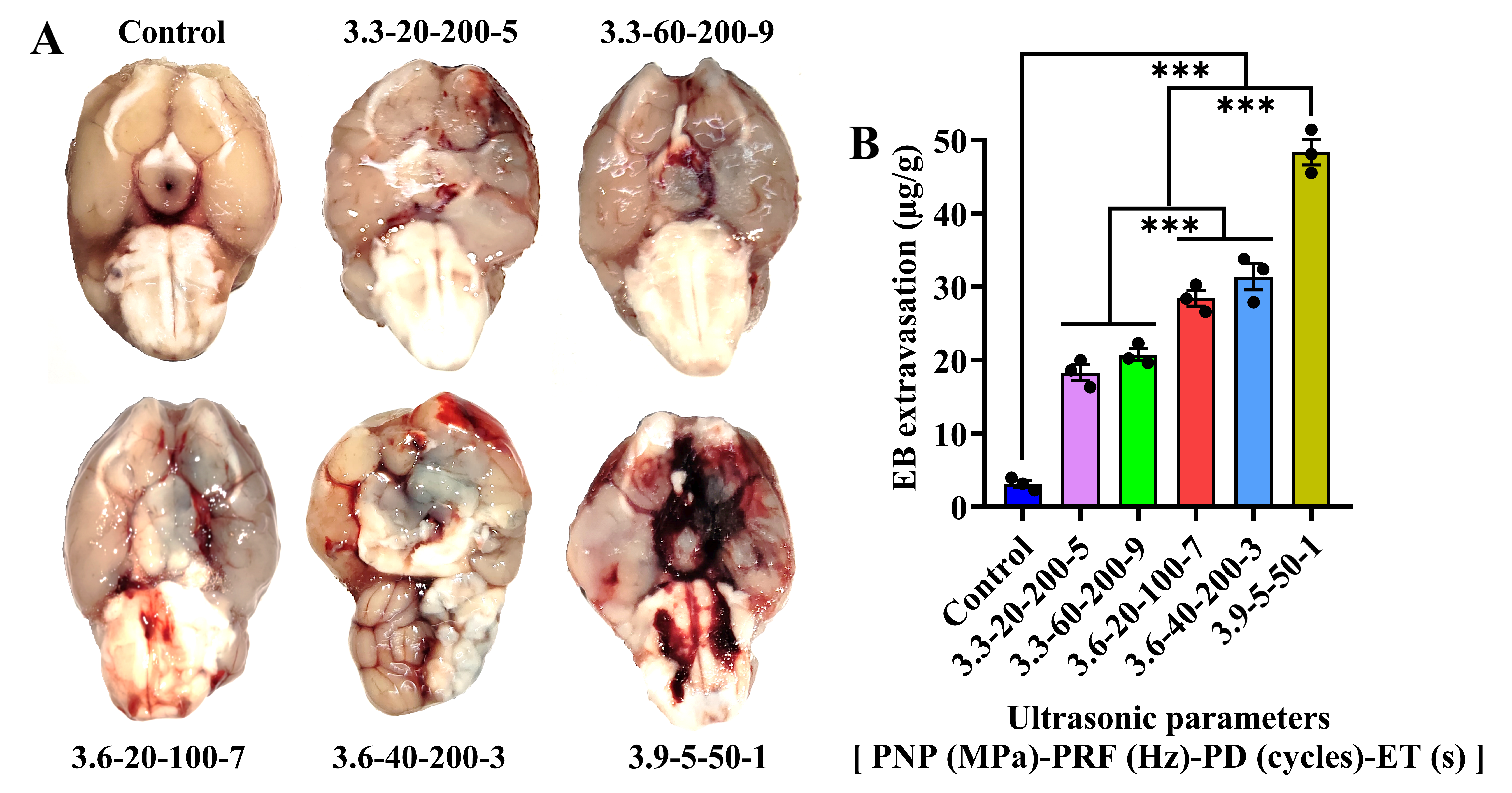

Supplement: Supplementary file 8 — Supplementary Material 8 [file 13346_2025_1828_MOESM8_ESM.tiff]

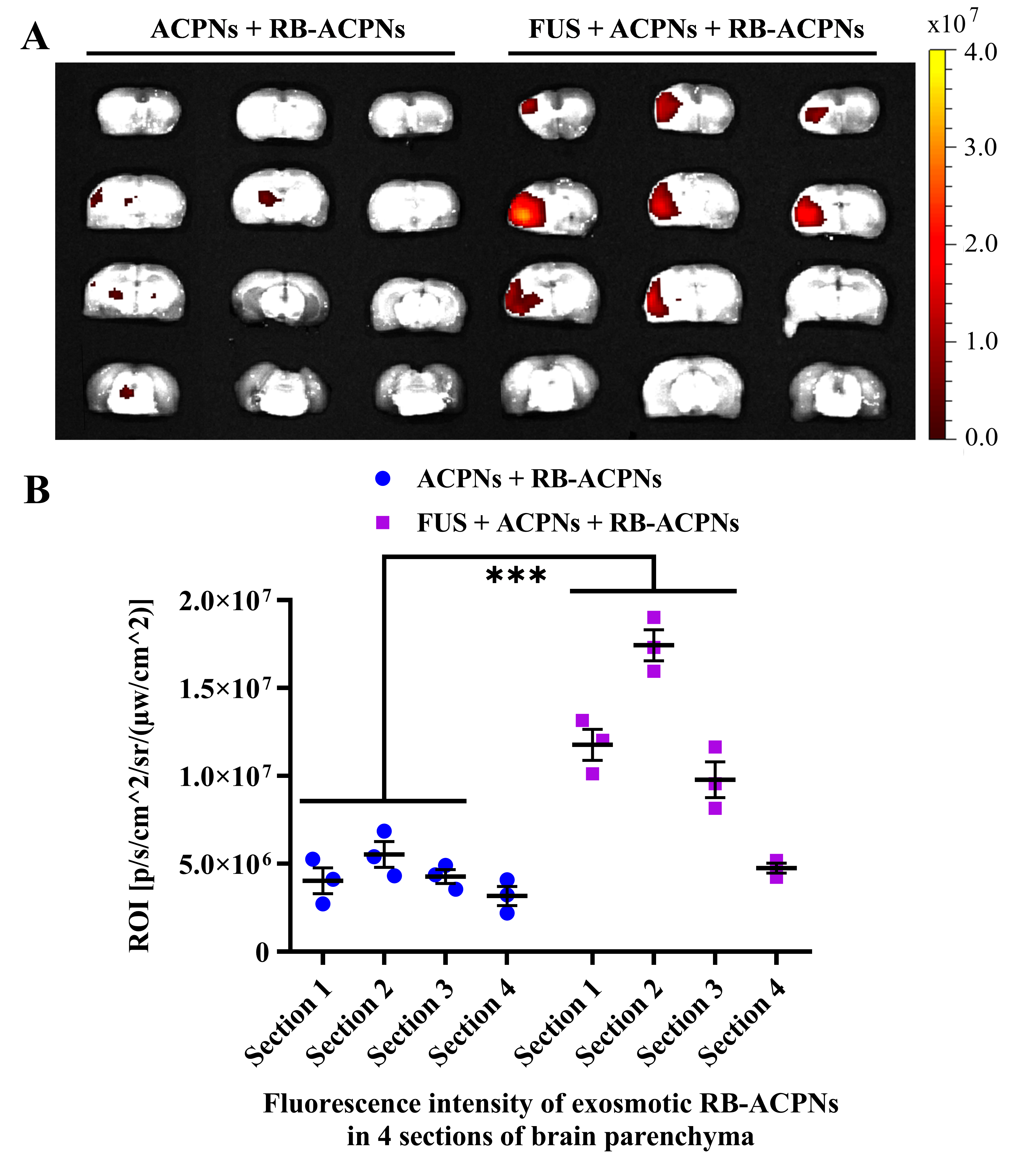

Supplement: Supplementary file 9 — Supplementary Material 9 [file 13346_2025_1828_MOESM9_ESM.tiff]

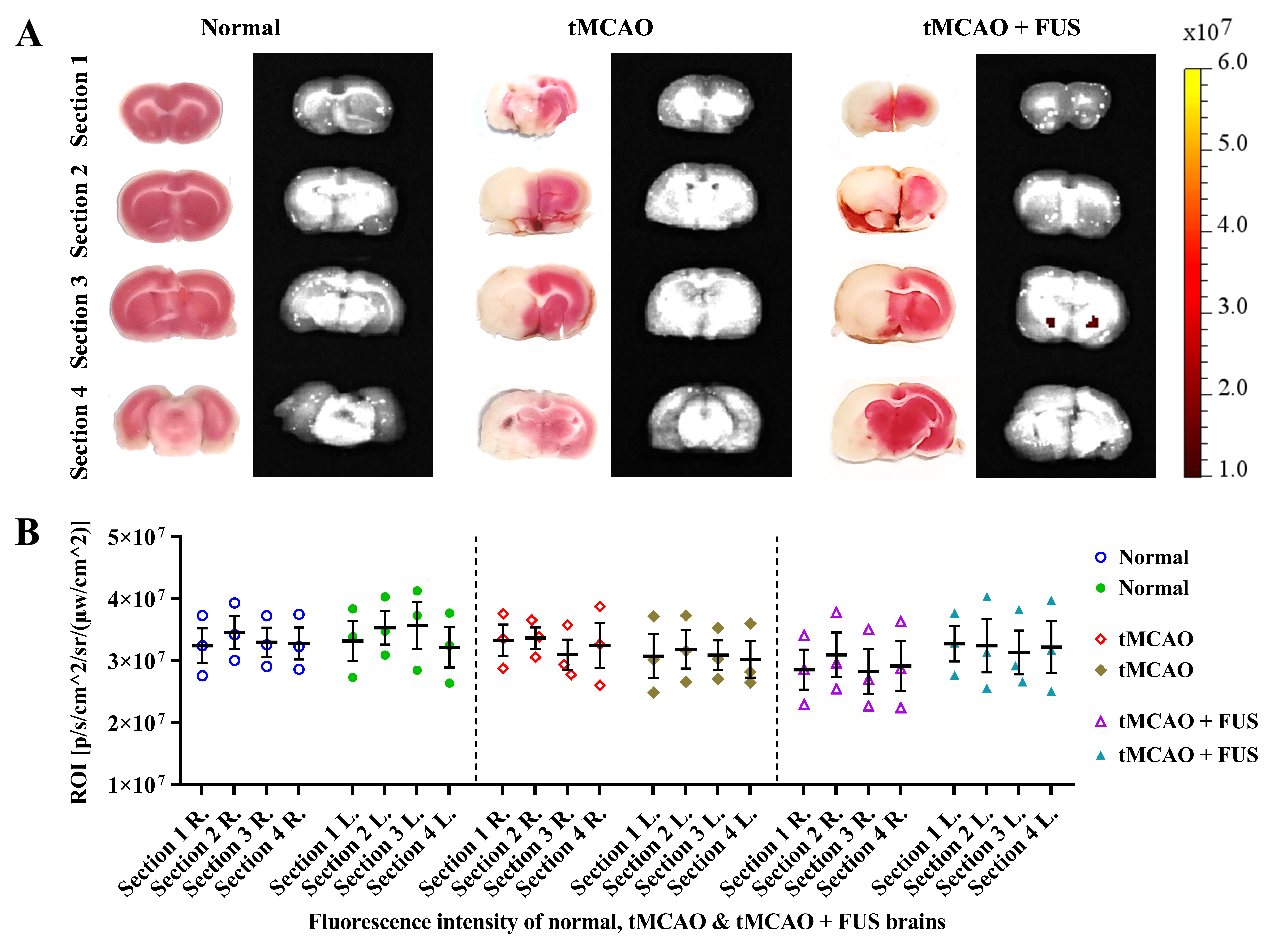

Supplement: Supplementary file 10 — Supplementary Material 10 [file 13346_2025_1828_MOESM10_ESM.tiff]

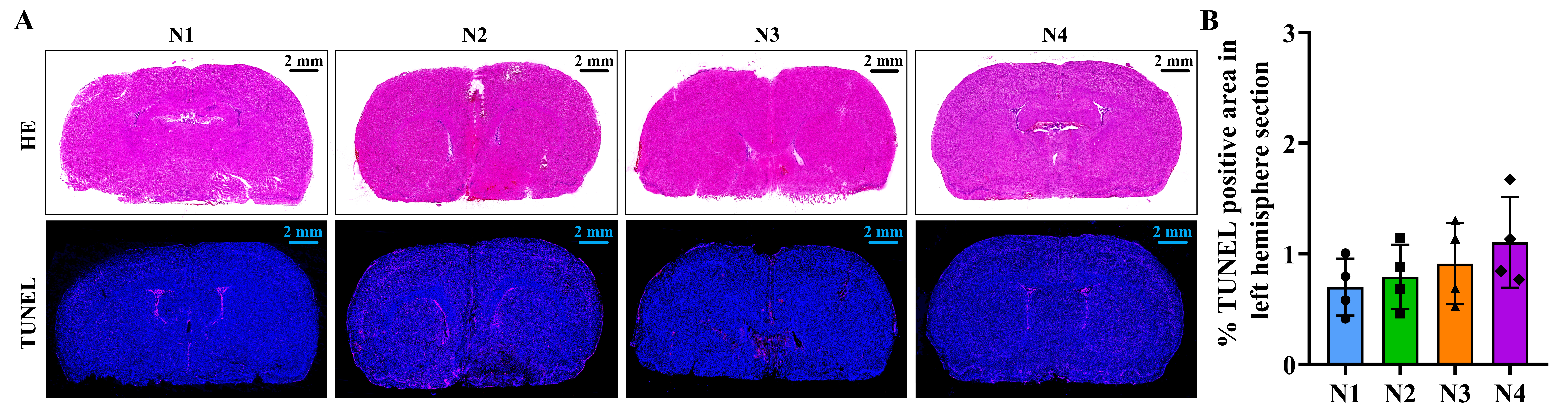

Supplement: Supplementary file 11 — Supplementary Material 11 [file 13346_2025_1828_MOESM11_ESM.tiff]
